# Comparison of Downstream Processing of Nanocrystalline Solid Dispersion and Nanosuspension of Diclofenac Acid to Develop Solid Oral Dosage Form

**DOI:** 10.3390/pharmaceutics12111015

**Published:** 2020-10-23

**Authors:** Sanika Jadhav, Amanpreet Kaur, Arvind Kumar Bansal

**Affiliations:** Solid State Pharmaceutics Lab, Department of Pharmaceutics, National Institute of Pharmaceutical Education and Research (NIPER), S.A.S. Nagar, Mohali, Punjab 160062, India; sanikaj17@gmail.com (S.J.); amanchahal027@gmail.com (A.K.)

**Keywords:** diclofenac acid, NanoCrySP, nanocrystalline solid dispersion, nanosuspension, downstream processing, fluidized bed processor, innovation radar, discriminatory dissolution, wet media milling

## Abstract

The conventional “top-down”, “bottom-up” and “combination” approaches of generating drug nanocrystals produce a “nanosuspension” (NS). It requires significant downstream processing for drying the liquid by suitable means followed by its granulation to develop an oral solid dosage form (OSD). In this paper, we used a novel, spray drying-based NanoCrySP technology for the generation of drug nanocrystals in the form of nanocrystalline solid dispersion (NCSD). We hypothesized that the NCSD would require minimal downstream processing since the nanocrystals are obtained in powder form during spray drying. We further compared downstream processing of NS and NCSD of diclofenac acid (DCF) prepared by wet media milling and NanoCrySP technology, respectively. The NS and NCSD were characterized for crystallinity, crystal size, assay and dissolution. The NCSD was physically mixed with 0.3% Aerosil^®^ 200, 1.76% croscarmellose sodium (CCS) and 0.4% sodium stearyl fumarate (SSF) and filled into size 0 hard gelatin capsules. The NS was first wet granulated using Pearlitol^®^ SD 200 (G1 granules) and Celphere^®^ 203 (G2 granules) in a fluidized bed processor, and the resulting granules were mixed using the same extra granular excipients as NCSD and filled into capsules. A discriminatory dissolution method was developed to monitor changes in dissolution behavior due to crystal growth during processing. Cost analysis and comparison of process efficiency was performed using an innovation radar tool. The NS and NCSD were successfully fabricated with a crystal size of 363 ± 21.87 and 361.61 ± 11.78, respectively. In comparison to NCSD-based capsules (65.13%), the G1 and G2 granules showed crystal growth and decrease in dissolution to 52.68% and 48.37%, respectively, in 120 min. The overall cost for downstream processing of NCSD was up to 80% lower than that of NS. An innovation radar tool also concluded that the one-step NanoCrySP technology was more efficient and required less downstream processing than the two-step wet media milling approach for conversion of nanocrystals to OSD.

## 1. Introduction

Poor aqueous solubility profoundly affects oral bioavailability of drugs belonging to Class II and IV of Biopharmaceutical Classification System (BCS). A plethora of strategies such as particle size reduction, salt formation, solvent mixtures, inclusion compounds and complexation have been employed to improve solubility [1]. Amongst these approaches, particle size reduction to nano range is a promising strategy to tackle solubility as well as dissolution rate limited oral bioavailability [2,3]. Emerging technologies offer a variety of opportunities for nanocrystal generation of poorly water-soluble drugs [1,2,4].

Drug nanocrystals are produced using “top-down”, “bottom-up” or “combination” technologies [5,6,7,8,9,10,11]. The product obtained using these approaches is a liquid dispersion of nanocrystals—i.e., a nanosuspension [1,12,13]. It possesses inherent physical and chemical instability issues such as sedimentation, crystal growth due to Ostwald ripening, solid-state transformation and hydrolysis. Moreover, due to use of aqueous media (to suspend poorly soluble drug or use as anti-solvent), nanosuspensions are prone to microbial growth upon storage [14,15,16,17]. On the other hand, the oral solid products are more patient compliant, relatively stable and hold a large market share [1,7]. Thus, incorporation of nanocrystals to oral solid dosage forms (OSDs) is essential for their commercial development [2,15,18].

The production of OSDs, without compromising physical, chemical and pharmaceutical properties of drug nanocrystals, is crucial [5]. Development of OSDs from nanosuspension primarily involves two steps—(i) drying of nanosuspension and (ii) incorporation of solidified nanosuspension to OSDs. Optimization of formulation components and process conditions is required for removal of the aqueous phase from the nanosuspension [14,15,19]. The main challenge during the drying process of nanosuspension lies in preserving the redispersibility of nanocrystals [1,20,21]. The performance attributes of nanocrystals critical during downstream processing include particle size, drug: excipient ratio, rate and extent of dissolution [15,16,22,23,24,25]. Factors such as ratio of extragranular excipients, lubricants, amount and solubility of diluents play a significant role in achieving the desired dissolution profile of solidified nanosuspension-based OSDs [22]. Furthermore, the downstream process should be efficient considering the cost, number of unit processes and final product efficacy [26,27]. Hence, development of an efficient downstream process that preserves the dissolution advantage of nanocrystals is necessary [19,25,28].

NanoCrySP is a novel “bottom-up” spray drying-based technique for the generation of nanocrystalline solid dispersion (NCSD) of poorly soluble drugs [29,30]. Drug and excipients are dissolved in a solvent system in a specific ratio, and then the solution is spray dried. A dry powder is obtained, wherein drug nanocrystals are embedded into the matrix of crystallization-inducing excipient and a stabilizer. Discrete primary particles of size 0.5 to 20 μm containing drug nanocrystals of size 10 to 1000 nm are thus, produced directly in powder form [29]. Hence, NanoCrySP technology is envisaged to require minimal downstream processing to formulate OSDs without compromising the dissolution benefits of nanocrystals [29].

For the present work, DCF (2-(2-(2, 6-dichloroanilino) phenyl) acetic acid) was chosen as a model drug. It is a non-steroidal anti-inflammatory drug (NSAID) with anti-inflammatory, analgesic and anti-pyretic activity [31]. The US Food and Drug administration (FDA) recommends that NSAIDs be used at the lowest effective dosage for the shortest duration to minimize gastro-intestinal, cardiovascular and renal adverse effects associated with NSAIDs [32,33,34]. The biological half-life of DCF is relatively small (2 h), and due to first-pass metabolism, only about 50% of the absorbed dose is systemically available. Moreover, DCF is a poorly water soluble (6.06 µg/mL) BCS class II compound with a pH-dependent solubility profile having a pKa of 4.18 and logP of 3.03 [35,36]. Thus, it requires improvement in solubility and dissolution rate to increase oral bioavailability at a minimal dose. Nanocrystals are a promising formulation approach to address this issue. Increase in solubility and dissolution rate by nanocrystals shall assist in rapid onset of action to enhance its therapeutic efficacy.

Iroko Pharmaceuticals LLC developed and commercialized oral capsules of DCF in the US market under the brand name ZORVOLEX^®^ in two different strengths—i.e., 18 and 35 mg. The capsules are produced using a patented dry-milling process—SoluMatrix Fine Particle Technology™—that grinds pharmaceutical drugs directly into a fine powder to the submicron level—10 to 200 times smaller than conventional drug particles [34]. Various literature studies also report generation of DCF nanocrystals in the form of nanosuspension to improve its oral bioavailability [37,38]. Nonetheless, additional unit operations necessary to incorporate nanosuspension into a patient compliant, physically stable OSD affect process efficiency of the technique. In contrast, NanoCrySP technology would produce DCF nanocrystals directly in powder form. Therefore, the downstream processing required for DCF nanocrystals produced via different techniques would significantly differ.

The objective of this work was to compare bottom-up NanoCrySP technology and the top-down wet media milling approach to thoroughly understand the efficiency of each process in developing nanocrystal-based OSDs. Size-based discriminatory dissolution studies, cost analysis and comparison of process efficiency using an innovation radar tool were used to highlight the complexities during downstream processing. A comparative evaluation of process efficiency of new technology with an established technology for nanocrystal generation has not been performed. The current work compares the process efficiency of the two methods for generation of nanocrystals on the basis of formulation, process attributes and cost. The present work shall create evidence for minimal downstream processing required for NCSD, thus opening translational avenues for the development of solid dosage forms of poorly water-soluble drugs using NanoCrySP technology.

## 2. Materials and Methods

### 2.1. Materials

Diclofenac acid (DCF) was procured as a gift sample from Amoli Organics Pvt. Ltd., Vadodara, India. Pearlitol^®^ SD 200 and Celphere^®^ 203 were received as gift samples from Ind Swift Labs Ltd., Panchkula, India and Signet Chemical Corporation, Mumbai, India, respectively. Mannitol (MAN), hydroxy propyl methyl cellulose LV E5 (HPMC LVE5), dioctyl sulfosuccinate sodium (DOSS) and sodium lauryl sulphate (SLS; Kolliphor^®^ fine) were received as gift samples from Windlas Biotech Pvt. Ltd., Dehradun, India. Sugars, polyols and amino acids were purchased from Himedia, Mumbai, India. Size 0 hard gelatin capsules were received as a free sample from ACG Capsules, Associated Capsules Pvt. Ltd. (ACPL), Mumbai, India. Organic solvents used were of analytical grade.

### 2.2. Methods

#### 2.2.1. Analytical Method Development of DCF Using HPLC

DCF was quantified using High Performance Liquid Chromatography (HPLC) (Chromatograph, Alliance 2690, Waters, Milford, CT, USA), equipped with an auto sampler (2707), a degasser (DG II), a photodiode array detector (2998) and an interface (Empower 2). The HPLC method was developed in-house using an RP-18e column (C-18 Lichrocart^®^ 100, 250 mm × 4.6 mm; 5 µm) (Merck, Darmstadt, Germany) for separation at 40 °C. A mobile phase comprising Acetonitrile: Citrate buffer (pH: 3) in ratio 70:30 *v*/*v* was run in isocratic mode at a flow rate of 1 mL/min. At an injection volume of 20 µL, DCF eluted through the stationary phase at a retention time of 5.6 min, and absorbance was taken at a wavelength of 280 nm.

A linear calibration curve was obtained in a concentration range of 2.5 to 160 µg/mL with a coefficient of correlation (*R*^2^) of 0.999. The HPLC method was validated for intra-/interday accuracy, repeatability, limit of detection (LOD) and limit of quantification (LOQ). The intra-/interday accuracy was confirmed by % recovery of 100% ± 1%. The coefficient of variation was less than 2% for intra-/interday repeatability. The LOD and LOQ were 0.42 and 1.28 μg/mL, respectively.

#### 2.2.2. Generation of NCSD of DCF Using NanoCrySP Technology

The selection of appropriate crystallization-inducing excipient, stabilizer and spray drying solvent system is important in NanoCrySP technology [29]. Several criteria were considered while selecting crystallization-inducing excipient such as, Generally Regarded As Safe (GRAS) status, fast crystallization kinetics, ability to induce crystallization of DCF during spray drying and concentration within Inactive Ingredients Database (IID). Sugars (glucose, lactose and dextrose), sugar alcohols (mannitol, sorbitol) and amino acids (alanine, threonine) were screened for this purpose. The stabilizer was selected by a visual screening method considering the wetting time of DCF in aqueous stabilizer solutions (20 mL). A fixed amount (5 mg) of DCF was dropped manually into aqueous solution of a surfactant. The wetting or sinking tendency and the time taken by the drug to drop down in the solution was noted. SLS, DOSS, Polysorbate 80, Poloxamer 407, Lecithin, hydroxy propyl cellulose (HPC), HPMC LVE5 and their binary and ternary combinations within a varying concentration range of 0.05–0.1% were examined.

A solvent system for spray drying was selected on the basis of the following criteria: solubility of DCF and excipients, low boiling point and suitability for spray drying, safety class and limit reported in ICH Q3C guidelines [39]. Different ratios (7:3, 8:2, 9:1, 4:3:3, 5:2:3 and 6:1:3) of organic (methanol, ethanol, acetone, isopropyl alcohol, tetrahydrofuran and ethyl acetate) and aqueous phase, to solubilize DCF and excipients, were scrutinized to prepare a stable feed solution for spray drying. The different proportions of DCF, crystallization-inducing excipient and stabilizers were dissolved within a total solid content range of 0.5–2% *w*/*v* with drug loading varying from 20% to 40%. To prepare the feed solution, DCF was dissolved in the organic phase while excipients were dissolved in water, followed by its addition to the organic phase. Hold-up studies of feed solution were carried out at room temperature to confirm the absence of precipitation till 6 h.

The NCSD was generated by dissolving 200 mg of DCF in 80 mL isopropyl alcohol (IPA) (a 0.5% *w*/*v* solution containing drug loading of 40% *w*/*w*); 287.5 mg MAN and 12.5 mg of SLS were dissolved in 20 mL water. The aqueous phase was then slowly added to the organic phase with continuous stirring to prepare a feed solution. The solution was spray dried using a laboratory scale spray dryer (LU228 model, Labultima Ltd., Mumbai, India) using optimized parameters—inlet air temperature of 100 °C with an outlet temperature of 45–50 °C, atomization pressure of 1.2 kg/cm^2^, feed flow rate of 3 mL/min, vacuum of 95–105 mm of water column and spray nozzle with an internal diameter of 0.7 mm in co-current spray mode. The product was collected and stored in transparent screw-capped glass vials in a desiccator until used further for characterization.

#### 2.2.3. Characterization of NCSD

##### Differential Scanning Calorimetry (DSC)

The samples (2–3 mg) were weighed directly in Tzero aluminum pan and thermal scans were recorded at a heating rate of 20 °C/min from 0 to 190 °C using DSC (Q2000, TA instruments, New Castle, DE, USA). DSC heating curves were analyzed using Universal Analysis^®^ (v4.5, TA Instruments, New Castle, DE, USA, 2000) software. Throughout the experiments, nitrogen gas purge was maintained at a flow rate of 50 mL/min. High purity standard of indium was used for calibration of instrument for temperature and heat flow. All measurements were carried out in triplicate.

##### Powder X-ray Diffraction (PXRD)

X-ray diffractogram of samples were recorded at a room temperature using PXRD (Rigaku Ultima IV diffractometer, Rigaku Corporation, Tokyo, Japan) operated at 40 kV and 40 mA power settings. About 300 mg of NCSD was placed in a polymethyl methacrylate sample holder, and analysis was carried out in a continuous scan mode with a step size of 0.01° over 1 sec in range of 3° to 50° 2θ values. The diffraction patterns were analyzed using OriginPro version 9.1 software (OriginLab Corporation, Massachusetts, MA, USA, 2018).

##### Size Determination Using Zetasizer^®^

The size of DCF nanocrystals was determined by dynamic light scattering using Zetasizer^®^ (Nano ZS, Malvern PANalytical, Worcestershire, UK) as per protocol reported in our previous studies [40]. Around 2.5 mg NCSD was dispersed in 10 mL of 0.1% *w*/*v* HPMC LV E5, 0.06% *w*/*v* DOSS and 0.03% *w*/*v* SLS containing aqueous medium. The dispersion was vortexed for 5 min followed by bath sonication (Power sonic 510, Hwashin Technology Co., Seoul, Korea) for 10 min, and the sample was analyzed immediately using disposable polystyrene cuvette in triplicates. For each sample, D90 (nm) and PDI were reported. The viscosity and refractive index of the dispersant media were 1.28 cP and 1.330, respectively.

##### Scanning Electron Microscopy (SEM)

The morphology and surface characteristics of NCSD particles were analyzed using SEM (S-3400, Hitachi Ltd., Tokyo, Japan) operated at an excitation voltage of at 10 kV. The sample was spread on a steel stage lined with double-sided adhesive tape and gold coated using an ion sputter (E-1010, Hitachi Ltd., Japan) before analysis.

##### Assay

The NCSD (87.5 mg equivalent to 35 mg of DCF) was dissolved in 10 mL IPA and water mixture in ratio 8:2. The standard stock was prepared using a physical mixture of DCF, MAN and SLS in ratio 40:57.5:2.5 and dissolved in 10 mL IPA and water mixture (8:2). Both standard stock and sample mixtures were sonicated using bath sonicator (Power sonic 510, Korea) for 5 min to obtain a clear solution. Both the stock and sample solutions were filtered through 0.1 μm nylon disc filters and analyzed using the HPLC method.

##### Determination of % Yield

The quantity of NCSD collected in the cyclone separator of spray drying assembly was used to measure the % Yield. The formula used to calculate % Yield was as follows: product weight recovered from cyclone/theoretical weight of solid as per volume of the solution sprayed × 100.

##### Moisture Content Determination

NCSD samples were accurately weighed (about 500 mg), and the moisture content was determined by Karl Fischer (KF) titration (Metrohm 794 basic titrino, Herisau, Switzerland). Disodium tartrate dehydrate was used for the calibration of the instrument. Moisture content of each sample was estimated in triplicates for accurate measurement.

##### Flow Properties

The bulk density of NCSD was determined using the cylinder method. A 50 mL measuring cylinder was filled approximately to 60% of its volume. The volume occupied by the sample in the measuring cylinder and weight of the sample was recorded. The tapped density of the NCSD sample was determined using the tapped density apparatus (ETD-1020, Tap Density Tester, Electrolab, Mumbai, India) in USP 1 mode. The measuring cylinder was fitted onto the tapped density apparatus and subjected to 500 and 1250 automated taps. Furthermore, volume of powder in cylinder after 500 (V500) and 1250 taps (V1250) was noted down. Carr’s index and Hausner ratio were calculated using bulk and tapped density values. Angle of repose of the NCSD powder sample was determined using the manual fixed funnel height method [41]. Flow properties were determined in triplicate, and the result was reported as mean ± standard deviation (SD).

#### 2.2.4. Generation and Characterization of DCF Nanocrystals Prepared by Wet Media Milling

Nanosuspension of DCF was prepared using wet media milling. An aqueous solution of SLS (0.1% *w*/*v*) and HPC (1% *w*/*v*) was used as dispersing and stabilizing media for preparation of nanosuspension. A 3 mL sample of this medium was taken in a 10 mL vial with a screw cap, and 150 mg DCF was dispersed in it to obtain a total drug loading of 5% *w*/*v*. The mixture was stirred at 6000 rpm using a magnetic bead for 5 min. Furthermore, 8 g of milling glass beads of size 0.1–0.3 mm were added and kept for stirring at 1500 rpm for 6–8 h. The resulting nanosuspension (NS1) consisted of DCF nanocrystals suspended in an aqueous stabilizer solution of SLS and HPC.

Particle size using Zetasizer^®^ and assay of NS1 were determined as per procedures described in Section 2.2.3.

#### 2.2.5. Wet Granulation Using Nanosuspension

NS1 was used as a granulating fluid for wet granulation of excipients, using a top-spray fluidized bed processor (FBP) (Mini Glatt, Glatt GmbH Process Technology, Binzen, Germany). Pearlitol^®^ SD 200 (particle diameter: 200 µm) and Celphere^®^ (Microcrystalline Cellulose (MCC)) 203 (particle diameter: 280 µm) were used as granulating substrates. The process parameters used were: inlet and bed temperature of 60 and 35 °C, respectively; atomization air pressure: 0.42 MPa; fluidization air pressure: 1.03 MPa. NS1 was top sprayed using a spray gun with a nozzle diameter of 0.3 mm. A 200 g sample of granulating substrate—i.e., Pearlitol^®^ SD 200 or Celphere^®^ 203 was granulated with 400 mL NS1 (with 20 g DCF) using 1.4 mL/min spray rate to obtain 10% *w*/*w* drug loading in the resulting G1 (Pearlitol^®^ SD 200) or G2 (Celphere^®^ 203) granules, respectively. Two different types of granulating substrates were employed to understand the impact of the nature of granulating substrate on the process.

#### 2.2.6. Characterization of Granules

Optical microscopy was utilized to visualize physical state of granules. Around 3–4 mg G1/G2 granules were mounted on a clean glass slide. Microscopic images of granules were captured using a microscope (Leica Microsystems, Wetzlar GmbH, Germany), in optical mode under 50× magnification. Images were captured using a DC 300 video camera (JVC, Linkam, Wetzlar, Germany) and analyzed using Linksys 32 software. The size of individual particles was measured, and D90 values of ≈200 particles as per number-based size distribution were reported. The difference in particle size before/after granulation and aggregation present in granules was analyzed in optical microscope.

Granules were characterized using SEM and PXRD using the method described in Section 2.2.3. SEM analysis helped to understand the surface changes upon granulation. PXRD analysis was performed to confirm crystallinity of DCF in G1 and G2 granules. The percent assay was calculated using the validated HPLC method. Additionally, the % Yield of granules was calculated as per the method described in Section 2.2.3.

#### 2.2.7. Incorporation of Nanocrystals to Solid Oral Dosage Form

Nanocrystals generated using NanoCrySP technology and the wet media milling method were incorporated into an OSD. Dosage form was chosen according to drug loading and the dose of the drug. Excipients were evaluated depending on the type of dosage form and flow properties of the blend. Various excipients such as Celphere^®^ 203/Pearlitol^®^ SD 200, croscarmellose sodium and Aerosil^®^ 200 were sieved through BSS #40 individually, and sodium stearyl fumarate was sieved through BSS #60 and then mixed with either NCSD1 or G1/G2. Flow properties such as bulk density, tapped density, angle of repose and Carr’s index were determined for powder blends as per the method described in Section 2.2.3. Capsules were formulated as per the formula described in Table 1; Table 2 wherein the same excipients were used for both the approaches. These blends were then filled into a size 0 hard gelatin capsules manually. Disintegration time of capsules was also determined using USP tablet disintegration apparatus (ETD-2L Disintegration Tester USP, Electrolab, India). Briefly, a single capsule was placed into distilled water media (maintained at 37.5 °C) filled in the basket rack assembly along with a disc to prevent floating of the capsule. The apparatus was operated at 32 cycles per minute.

It should be noted that in the case of NanoCrySP technology, NCSD1 was obtained in a powder form and thus, only physical mixing with excipients was required to formulate OSD. NS1 (generated using wet media milling as given in Section 2.2.4.) was used for wet granulation, and then OSDs were formulated by mixing extragranular excipients with either G1 or G2 granules. A formulation was also generated using micronized DCF by blending it with excipients, using the same manufacturing procedure as that of NCSD1.

#### 2.2.8. Discriminatory Drug Dissolution Studies

A dissolution method was developed to discriminate dissolution profiles of DCF nanosuspensions of different particle sizes. Dissolution profiles of NCSD1 and G1/G2 capsules were compared to investigate the impact of downstream processing on DCF nanocrystals’ size. USP Type II dissolution apparatus (TDT-08L, LabUltima, Mumbai, India) containing 1000 mL citrate buffer (pH: 5.5) medium was maintained at a temperature of 37 ± 0.5 °C and 50 rpm. Capsules were introduced in a dissolution jar along with sinkers. Samples (2 mL) were withdrawn at 5, 7.5, 10, 12.5, 15, 17.5, 20, 30, 45, 60, 90 and 120 min and filtered through 0.1 μm nylon disc filter paper. Samples were immediately diluted with acetonitrile in ratio 1:1 and analyzed using the developed HPLC method. At each time point, fresh medium was added to keep the volume of dissolution media constant. Percent DCF dissolved with time was reported as the mean value ± standard deviation (SD). The dissolution profiles were compared using a model-independent approach using similarity factor (F2) by DDSolver add-in program. Dissolution studies were performed in triplicate to ensure the reproducibility of results.

#### 2.2.9. Stability of Intermediate Products

Accelerated stability studies of NCSD1, packed in double polyethylene-lined aluminum pouches, were carried out at 40 °C/75% RH for 3 months wherein samples were analyzed at intervals of 15, 30 and 90 days. Size of nanocrystals using Zetasizer^®^, assay, PXRD and dissolution were performed according to methods described in Section 2.2.3 and 2.2.8, respectively. NS1 was also kept in glass vials covered with aluminum foil for stability testing at 25 °C for 20 days. Size of DCF nanocrystals and assay were determined at time intervals of 5, 10 and 20 days as per methods described in Section 2.2.3. Dissolution profiles of stability samples were carried out as per the method discussed in Section 2.2.8 and compared with the initial sample.

#### 2.2.10. Comparison of Process Efficiency of NanoCrySP Technology and Conventional (Wet Media Milling Followed by Wet Granulation Using FBP) Approach

##### Costing of Both the Approaches

The cost of each process was determined at two stages. The cost required for the generation of nanocrystals was considered as the first stage, and the second stage was the incorporation of nanocrystals into oral solid dosage form. In the case of NanoCrySP technology, generation of NCSD1 and then its physical mixing with excipients comprised phase 1 and 2, respectively. While in the case of media milling approach, phase 1 comprised generation of NS1, and phase 2 further consisted of two-steps—(i) wet granulation of excipients using NS1 in FBP and (ii) physical mixing of G1/G2 with extra granular excipients, for capsule manufacturing.

The overall cost required for manufacturing 100,000 capsules was calculated, considering the cost of capital equipment, ingredients, manpower, electricity, product analysis and miscellaneous parameters. Manpower was calculated as a product of per day wage, number of working days and total employees. Similarly, a product of per day electricity cost and the number of days of electricity consumed resulted in total electricity cost. A comparison of the phase 2 cost of each methodology assisted in identifying the most cost-effective downstream process.

##### Comparison of Various Quantitative and Qualitative Aspects of Both the Processes

Different parameters such as the number of steps, solid form, yield, time, reproducibility and drug loading per batch were analyzed and compared for each approach. Quantification of various qualitative attributes was essential to compare process efficiency [42,43,44]. Hence, rating (1 to 5) was assigned to each attribute to convert into a quantifiable parameter. Similarly, it is important to give weightage (1 to 5) to each parameter depending upon the impact of a parameter on process efficiency and final product attributes [45]. The score (1 to 25) of each attribute was calculated by multiplying rating and weightage.

In addition to this, the statistical significance of percent drug dissolved with time from the discriminatory medium was assessed by calculating *p*-value using an independent *t*-test in MS Excel (Microsoft office, Washington, DC, USA) which helped to understand if the two processes were comparable. In the end, a comparison of rating and scores of two methods was depicted using the innovation radar tool.

## 3. Results

### 3.1. Generation of NCSDs

After screening various combinations of solvents, IPA: water in ratio 8:2 was selected as the spray drying solvent system (for details, refer to Supplementary Materials: Table S1). Visual examination of the solvent system confirmed absence of precipitation overnight at a solid content of 0.5% *w*/*v* and drug loading of 40% *w*/*w*. Moreover, the residual limit of IPA in the spray-dried product was 1500 ppm, which was within the safer limits (<5000 ppm). MAN assisted in generation of DCF nanocrystals in NCSD by providing sites for heterogeneous nucleation during spray drying (for more details, refer to Supplementary Materials: Section 1, Tables S2 and S3 and Figure S1). MAN was pursued further as the resulting NCSD1 had good flow properties (Carr’s index: 15.83). Furthermore, NCSD1 had low moisture content (0.27 ± 0.12) and highest % Yield (50 ± 5.06). This shall be advantageous for granulation of NCSD (refer to Table S4 of Supplementary Materials for more details). The dispersibility studies conferred that DCF dispersed quickly in SLS solution than other screened stabilizers (data shown in Table S5). Thus, NCSD1 was generated using DCF: MAN: SLS in the ratio of 40:57.5:2.5 with a solid content of 0.5% *w*/*v*.

### 3.2. Characterization of NCSD

**Figure 1:**
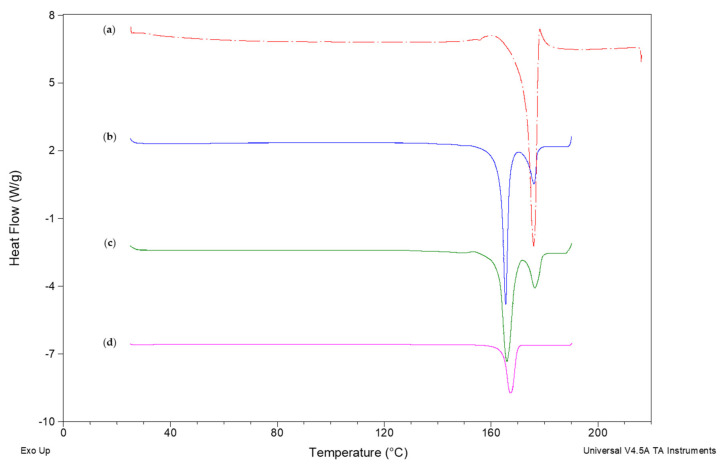
DSC heating curves of (**a**) DCF, (**b**) NCSD1, (**c**) physical mixture (PM) and (**d**) MAN showing melting point of DCF at 172.68 and 173.06 °C in pure DCF and NCSD1, respectively.

Figure 1 shows DSC thermograms of DCF, NCSD1, physical mixture (PM) of DCF and MAN (4:6) and MAN. DCF showed a melting endotherm at 175.97 °C; ΔH_f_—133.4 J/g, whereas MAN showed melting point at 167.10 °C; ΔH_f_—243.7 J/g. The heating curve of PM showed a melting point of DCF and MAN at 173.83 °C; ΔH_f_—28.69 J/g and 165.88 °C; ΔH_f_—120.5 J/g, respectively. In the case of NCSD1, the endothermic peaks corresponding to the melting point of crystalline DCF and MAN were observed at 176.19 °C; ΔH_f_—26.37 J/g and 165.54 °C; ΔH_f_—125.3 J/g (midpoint), respectively (refer to Figure 1). No T_g_ was observed during the heating cycle in NCSD1, thus confirming the crystalline state of DCF.

**Figure 2:**
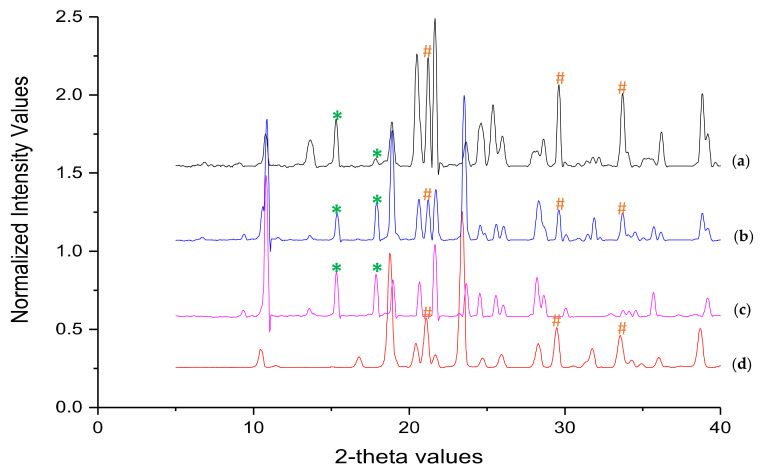
Overlay of PXRD pattern of (**a**) NCSD1 (**b**) PM, (**c**) DCF and (**d**) MAN. The characteristic peaks of DCF and MAN have been marked with * and #, respectively.

Figure 2 shows overlay of diffraction patterns of DCF, MAN, PM of MAN and DCF and NCSD1. The characteristic peaks of Form II of DCF were observed in NCSD1 at 2θ values of 15.36° and 17.92°. This further confirmed the crystalline nature of DCF in NCSD1. The other characteristic peaks of Form II of DCF at 10.80°, 13.66°, 18.84°, 23.62°, 24.72° and 26.01° overlapped with that of MAN (not highlighted in figure). Furthermore, NCSD1 and PM showed the characteristic peaks of Form I, II and III of MAN at 21.22°, 29.55° and 33.62°.

**Figure 3:**
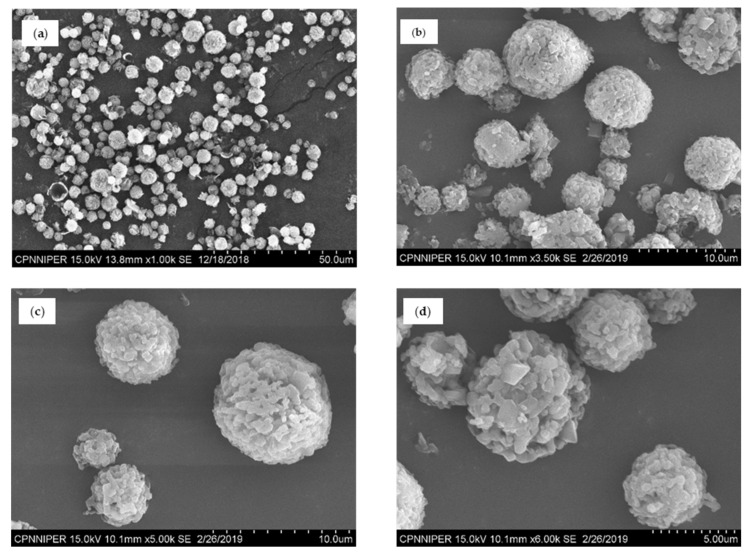
SEM images of primary particles of NCSD1 at scale 50 (**a**), 10 (**b**,**c**) and 5 (**d**) µm, revealing spherical particles.

SEM analysis of NCSD1 (Figure 3) revealed spherical but rough particles in the size range of 5–10 µm. DCF nanocrystals embedded in NCSD1 had D90 of 363 ± 21.87 nm (please refer to Table S6 in the Supplementary Materials). Moisture content in NCSD1 was 0.27%, which was statistically significant (*p*-value < 0.05) as compared to other prototypes (experimental values of other prototypes are given in Supplementary Materials: Table S7). Percent assay of NCSD1 was in the acceptable limits—i.e., 99.23%. Thus, NCSD1 was selected for development of NCSD-based OSD of DCF.

### 3.3. Generation and Characterization of DCF Nanocrystals Prepared by Wet Media Milling

The particle size of DCF crystals reduced to 361 nm in 6 h, and further milling did not reduce DCF crystal size significantly. The size of DCF nanocrystals after wet media milling is reported in Table 3. Percent assay of DCF in NS1 was 99.8%.

### 3.4. Wet Granulation Using Nanosuspension

#### 3.4.1. Impact of Wet Granulation Method

Considering a minimum batch size of 250 g in FBP, 5% *w*/*v* drug loading of nanosuspension and 35 mg dose of DCF, 400 mL NS1 was required for granulation of substrates. The same volume of NS1 could not be granulated in a rapid mixer granulator (RMG). As in the case of RMG, the whole quantity of NS1 was poured at a time, which led to liquefaction of the granulating substrate, Pearlitol^®^ SD 200. Thus, for the incorporation of nanosuspension to OSDs, wet granulation using FBP was the best suitable technique.

#### 3.4.2. Influence of Process Parameters

**Figure 4:**
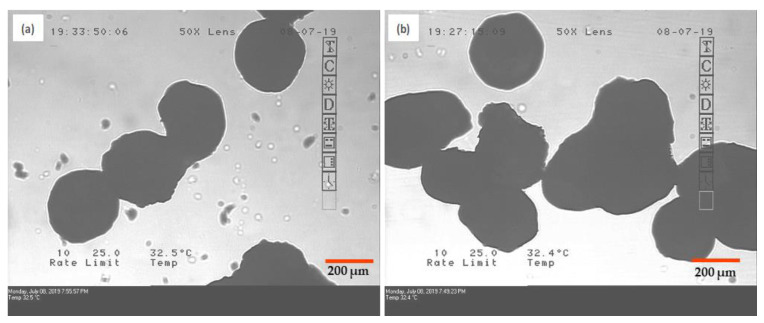
Microscopic images showing aggregation of granules in both the cases, (**a**) G1 and (**b**) G2, when spray rate was increased from 1.4 to 3 mL/min.

Process parameters in FBP have a significant impact on product attributes. Inlet temperature and feed spray rate are the most crucial process parameters [46,47]. Thus, when spray rate was increased from 1.4 to 3 mL/min and other parameters were kept constant, total processing time reduced to half but resulted in agglomeration in both G1 and G2 granules, due to insufficient drying time (refer to Figure 4). In other trials, keeping all other parameters constant, inlet temperature was increased up to 80 °C. This led to a reduction in the drying time, but the yield of granules decreased up to 20%. Thus, optimization of spray rate and inlet temperature was essential.

#### 3.4.3. Effect of Granulating Substrate

**Figure 5:**
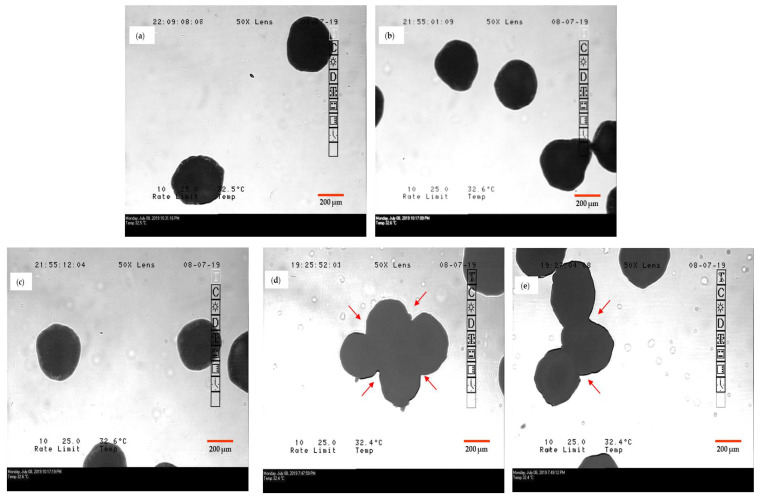
Microscopic images of (**a**) Pearlitol^®^ SD 200, (**b**) G1 granules, (**c**) Celphere^®^ 203 and (**d**,**e**) aggregated G2 granules marked by arrows in red.

Hydrophobicity of granulating substrate had a remarkable impact on the dissolution profile of the product, which is explained in the proceeding sections. Additionally, chances of over granulation with a slight increase in granulation time were high in the case of G2 granules. MCC has been reported to be more susceptible to over granulation with a slight increase in processing time [48]. This was confirmed experimentally by aggregation observed in microscopic images of over granulated G2 granules (Figure 5). On the contrary, increase in granulation time did not form aggregated particles in the case of Pearlitol^®^ SD200 containing G1 granules.

#### 3.4.4. Impact of Drug Loading of Nanosuspension on Wet Granulation

Nanosuspension with 1% *w*/*v* drug loading (NS5) required 2000 mL granulating fluid to achieve 10% *w*/*w* drug loading in granules. A large volume of granulating fluid could significantly increase the granulation-processing time. This could be reduced by increase in the drug loading of nanosuspension to 10% *w*/*v* (NS6). However, the increase in drug loading resulted in agglomeration, which was evident by higher crystal size (D90-1100 nm) of DCF nanocrystals in NS6. In the case of NS1, just 400 mL nanosuspension was required to achieve 10% *w*/*w* drug loading in granules, and this reduced the processing time as compared to NS5. Therefore, NS1 with 5% *w*/*v* drug loading was best suited considering the stability of nanosuspension as well as feasibility of wet granulation using FBP. Thus, drug concentration of nanosuspension was a critical parameter during wet granulation.

### 3.5. Characterization of FBP Processed Wet Granules

**Figure 6:**
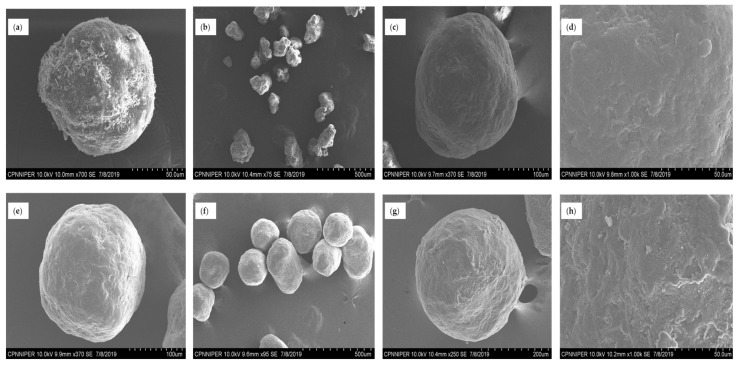
SEM images of (**a**) Pearlitol^®^ SD 200, (**b**) G1 granules, (**c**,**d**) magnified images of G1, (**e**) Celphere^®^ 203, (f) G2 granules and (**g**,**h**) magnified images of G2.

The size distribution of granulating substrates increased postgranulation as compared to the initial input material. D90 value for Pearlitol^®^ SD 200 increased from 183.18 µm to 220.20 µm postgranulation. Similarly, Celphere^®^ 203 showed an increase in D90 value from 282.50 µm to 342.21 µm on granulation. This confirmed successful granulation of Pearlitol^®^ SD 200 and Celphere^®^ 203 with NS1. The SEM images of granules pre- and postgranulation can be seen in Figure 6a–h. The particles had spherical morphology and smooth surface characteristics.

**Figure 7:**
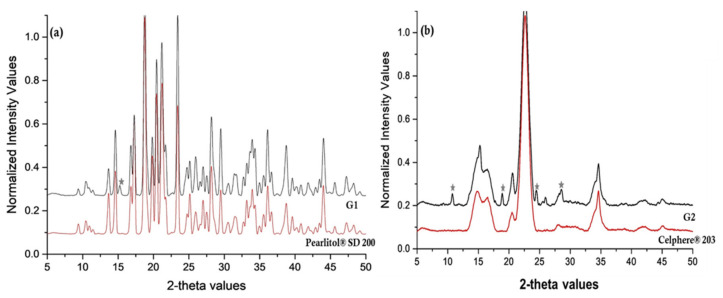
(**a**) Overlay of PXRD pattern of G1 and Pearlitol^®^ SD 200 showing characteristic peaks of DCF in G1 with *. (**b**) Overlay of PXRD pattern of G2 and Celphere^®^ 203. The characteristic peaks of DCF in G2 have been marked with *.

Percent assay of DCF in G1 and G2 granules was found to be 98.39% and 98.4%, respectively. Overlay of diffraction patterns of blank Pearlitol^®^ SD 200 and G1 granules is depicted in Figure 7a. In the case of the G1 sample, it was difficult to find distinguishable characteristic peaks of DCF as most of the peaks overlapped with Pearlitol^®^ SD 200. Non-interfering characteristic peaks of polymorph II of DCF were observed at 15.36°, while the rest of the peaks at 21.68°, 24.68° and 26.51° 2θ overlapped with MAN, in G1 granules. Furthermore, G2 granules revealed characteristic peaks of DCF-Form II at 2θ values of 10.80°, 18.94°, 24.66° and 28.63° (Figure 7b). Therefore, it was confirmed that DCF retained its crystalline form in granules, and no solid-state transformation occurred during wet granulation. The decreased intensities of characteristic peaks of DCF could be attributed to relatively low concentration of drug as compared to the granulating substrate.

The size of DCF nanocrystals in G1 and G2 granules could not be measured using Zetasizer^®^ (as per the method described in Section 2.2.3.) as Celphere^®^ swelled in the dispersion medium. Thus, discriminatory dissolution studies were performed to understand the impact of downstream processing on crystal size of DCF.

### 3.6. Formulation of Solid Oral Dosage Form: Effect of Dosage Form

A tablet as the dosage form was not considered as parameters such as compaction pressure, compressibility, hardness and tensile strength can affect product attributes. The tableting process can alleviate or aggravate downstream processing effects such as crystal growth, which could be misleading. As a result, a capsule was selected as the dosage form [1].

The granulated blends of NCSD1, G1 and G2 had good flow properties with Carr’s index and angle of repose in the range of 11–16% and 31–33°, respectively (refer to Supplementary Materials: Table S8 for more details). Disintegration time of NCSD1, G1 and G2 capsules was in the range of 2–2.5 min suggesting no effect of excipient or process on the disintegration of capsules.

### 3.7. Discriminatory Drug Dissolution Studies

#### 3.7.1. Development of Discriminatory Dissolution Medium

Non-sink medium with index close to 1 and slower rpm are reported to provide better discrimination of dissolution profiles due to change in particle size or solid form of a drug [49,50,51]. Generally, F2 values greater than 50 signify “sameness or equivalence” of the two profiles, and F2 values less than 50 indicate a “difference” in the two curves [52,53,54,55,56]. After screening different dissolution media, 1000 mL citrate buffer medium (pH 5.5) with a sink index of 0.8 and 50 rpm was selected as a discriminatory dissolution medium (refer to Supplementary Materials: Sections 2 and 3, Table S9 and Figure S2 for details about solubility study of DCF and screening of discriminatory dissolution medium). In this medium, a significant difference in % DCF dissolution was observed with time. NS2 and NS4 showed discriminatory dissolution profiles with an F2 value of 31.87. Dissolution profiles of NS2 and NS3 were also discriminatory with an F2 value of 44.77. Moreover, dissolution profiles of NS3 and NS4 were differentiable with an F2 value of 48.25. Hence, the media could detect variation in DCF particle size by virtue of differences in rate and extent of drug dissolution with time.

#### 3.7.2. Comparison of Dissolution Profiles

**Figure 8:**
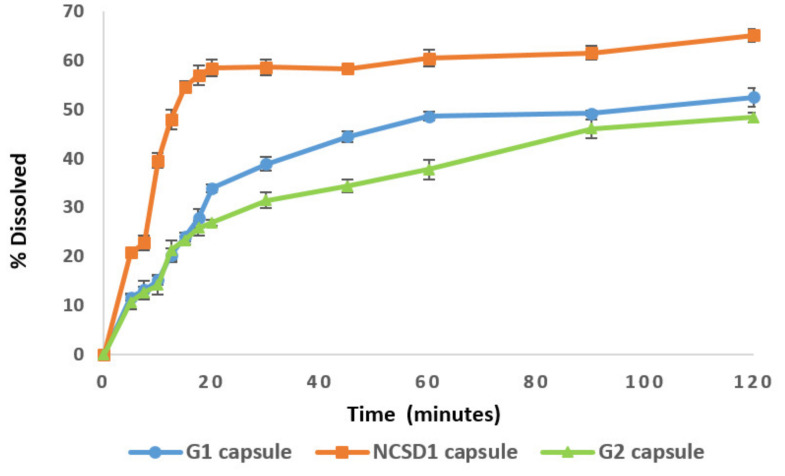
Comparison of dissolution profiles of NCSD1 and granulated (G1 and G2) nanosuspension-based capsules.

Dissolution of NCSD1, G1 and G2 granules in discriminatory medium after 120 min was 65.13%, 52.68% and 48.37%, respectively (Figure 8). Dissolution profiles of NCSD1 capsules and G1 capsules were found to be discriminatory with an F2 value of 35.48. Additionally, differentiable dissolution profiles were demonstrated for NCSD1 capsules and G2 capsules with an F2 value of 31.95. Discriminatory dissolution studies were used to detect change in DCF particle size after downstream processing. Thus, discriminatory dissolution of these three formulations pointed towards different DCF particle size in these formulations. A lower % dissolved from G1/G2 capsules as compared to NCSD1 capsules, could be possibly due to an increase in DCF nanocrystal size during downstream processing of NS1. NCSD was not subjected to significant downstream processing; thus, the chances of crystal growth were expected to be minimal. On the contrary, NS was wet granulated and dried from liquid medium, which poses chances of crystal growth due to agglomeration and aggregation during drying [57]. The drastic decrease in % DCF dissolved in the case of G1/G2 capsules signified the impact of downstream processing on size of DCF nanocrystals. Any crystal growth during downstream processing would supersede the dissolution advantage expected from size reduction.

Furthermore, the impact of excipient properties on dissolution was observed in the case of G1 and G2 capsules. The % drug dissolved with time in the case of G1 capsules (52.68%) was higher than that of G2 capsules (48.37%). This is because of differences in the mechanism of dispersibility of Pearlitol^®^ SD200 and Celphere^®^ 203 in water. Probable reasons for these results are discussed (refer to Supplementary Materials: Section 4).

### 3.8. Stability of Intermediate Products

PXRD analysis of NCSD1 stability samples confirmed crystalline nature of DCF and absence of polymorphic transformation of DCF over a period of 90 days (refer to Supplementary Materials: Figure S3). No increase in DCF nanocrystal size, in the case of NCSD1, was observed in 90 days (data shown in Table S10 of Supplementary Materials). Zetasizer^®^ analysis of NS1 showed no increase in DCF nanocrystal size in 20 days (see Supplementary Materials: Table S11). The assay of stability samples of both intermediate products was maintained in the range of 97–99%. The dissolution studies revealed no variation in percentage of drug dissolved with time in either NCSD1 or NS1 on storage (for more details, see Supplementary Materials: Figures S4 and S5).

### 3.9. Comparison of Process Efficiency

#### 3.9.1. Costing

In the case of NanoCrySP technology, 9.18 kg NCSD1 (0.5% *w*/*v* solid content) was required to manufacture 100,000 capsules (refer to Table 4). As the yield of spray dryer was 50%, starting material equivalent to 18 kg NCSD1 was taken. All costing in this paper was performed in Indian Rupee (INR). The phase 1 and phase 2 costs for NanoCrySP technology were INR 1,469,644 and INR 200,000, respectively. Furthermore, the effect of solid content of NCSD on cost of NanoCrySP technology is discussed in Supplementary Materials: Section 5 and Table S12.

The output of the wet media milling method and FBP was 95% and 85%, respectively. So, 88 L of NS1 (10% *w*/*w* drug loading) was required to produce 35.57 kg granules containing 3.557 kg DCF nanocrystals to manufacture 100,000 capsules of G1/G2 granules (Table 5). The analytical cost of INR 50,000 was considered for both the approaches for analytical evaluation.

Phase 1 cost for NanoCrySP technology was higher as compared to the conventional approach. However, the comparison of phase 2 cost was crucial to decide the efficiency of downstream processing of nanocrystals. Phase 2 cost for NanoCrySP technology was approximately INR 183,683 for solid content of 0.5% *w*/*v*, whereas for media milling it was INR 954,210, despite 10% drug loading in NS1. Thus, the downstream processing cost for NanoCrySP technology was 80% lower than that of the conventional process (see Supplementary Materials: Table S13). This drastic difference in cost could be attributed to wet granulation required in the case of the NS1 to incorporate it into OSDs. The cost related to capital equipment, ingredients, manpower and electricity increased to perform this additional unit process. Furthermore, the number of days required for downstream processing of the conventional approach was around 11 days unlike 2–3 days for NanoCrySP technology.

The cost associated with a particular type of granulating substrate used in Phase 2 had an impact on the overall cost. The capsules manufactured using Pearlitol^®^ SD 200 had a comparatively lower price than those produced using Celphere^®^ 203 as the substrate (refer to Supplementary Materials: Table S13). Thus, considering phase 2 cost, NanoCrySP technology was more cost efficient as compared to the media milling process.

#### 3.9.2. Comparison of Various Attributes

A statistically significant difference (*p*-value–0.01) was observed in the dissolution profiles of NCSD1 and G1/G2 capsules in the developed discriminatory dissolution medium. Moreover, the dissolution from NCSD1 capsules was more uniform and consistent as compared to G1/G2 capsules.

Various qualitative and quantitative characteristics of both the processes are depicted in Supplementary Materials: Table S14. This representation assisted in understanding product attributes for a comparative analysis of the two processes in proceeding sections. Grading of rating, weightage and scores are presented in Tables S15 and S16, respectively. Table S17 of Supplementary Materials describes the categorization of each rating of divergent variables that helped to decide the rating for each parameter; a desirable characteristic was given a rating of 5.

Dissolution, phase 2 cost, number of steps to formulate OSDs and solid state of the final product were variables with a weightage of 5. This was mainly because outputs of these parameters were critical as they directly impacted final product properties and efficiency of downstream processing. Table 6 shows scores that helped to get final comparative values for both the approaches. No. of steps, dissolution, intermediate product stability, solid state of final product and phase 2 cost were the most highly scored attributes (score of 25) for NanoCrySP technology as they had desirable characteristics. Number of steps, yield and solid state of the final product were maximum scoring parameters for the conventional approach.

#### 3.9.3. Innovation Radar

This tool is used to measure innovativeness of any product/process or organization. Analysis of multidimensional aspects of product/process can be presented together in a pictorial way. Such pictorial comparison helps to plot and interpret a wide range of quantifiable aspects. Moreover, it allows separation of critical impact factors from many trivial parameters, and thus, the focused and effective conclusive action plan can be employed to stand out as a novel product/process from well-established competitors [58,59]. This strategy is mostly used for evaluation of a novel product/organization and has not been extensively used for pharmaceutical processes till now. Parameters to be considered to examine efficiency of product/process should be customized according to a particular product/process. In this paper, we applied this technique to compare NanoCrySP technology with a conventional approach.

**Figure 9:**
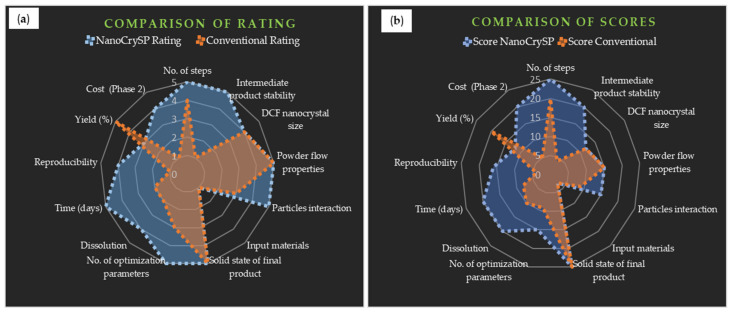
Comparison of (**a**) rating and (**b**) scores for NanoCrySP and conventional approach showing the area covered by each process.

The pictorial representation of comparative evaluation of process efficiency was possible using an innovation radar wherein various parameters with differing rating/scoring were plotted together for a particular process. The ideal innovation radar should cover the maximum area of the circle and should be equally distributed at all points [58,59]. Hence, the more uniformly distributed the radar, the more efficient the process. The radars representing a comparison of rating and scoring of both the processes are shown in Figure 9.

In the case of NanoCrySP technology, the radar of both rating and scores was closer to the periphery at points with maximum rated/scored attributes and occupied larger area as compared to the conventional approach. However, it was observed that the radar of rating occupied larger area than the radar of scoring for each approach. This difference amongst the two radar types was attributed to assignment of weightage in the case of score designation unlike rating. Thus, it was evident that a comparison of sole ratings could give misleading results, ignoring the consideration of critical attributes’ preference while determining process efficiency.

A comparison of radars of scores showed that area covered by NanoCrySP technology was larger and more uniformly distributed than that of the conventional approach. Hence, NanoCrySP technology has resulted in a more desirable process and product attributes compared to the latter.

## 4. Discussion

The techniques employed for generation of nanocrystals produce a liquid dispersion of nanocrystals—i.e., a nanosuspension (NS). Malamatari et al. reported various physical and chemical stability issues associated with NS, such as sedimentation, crystal growth, aggregation, solid-state transformation and hydrolysis. Moreover, due to the use of aqueous media, they are prone to microbial growth upon storage [15]. Considering these risks, the commercial viability of nanosuspensions pose a main challenge [14,15,16]. Hence, the development of a physicochemically stable and easy to scale-up nanocrystal-based OSDs is imperative [2,15].

The downstream processing of NS poses various challenges with respect to manufacturing of OSDs. Gao et al. reported that drying of NS can result in aggregation and eventually decreased dissolution kinetics of nanocrystals [1]. It can also cause drug degradation and decreased drug release due to additional thermal stresses used during the drying process [8]. Addition of a carrier or matrix former is thus essential to prevent drug aggregation during drying of NS [60]. Furthermore, maintaining optimal drug loading while incorporating a carrier is another pre-requisite [61]. The process parameters during drying of NS have an impact on the dissolution profile of dried nanosuspension [62]. This implies that there is a need for emergence of new technologies that significantly reduce the processing steps, risks (especially dissolution rate) and hasten the development of nanocrystal-based OSDs.

NanoCrySP technology employs a novel spray drying-based process that produces drug nanocrystals in powder form. The drug is spray dried from a solution in the presence of crystallization inducer, to produce NCSD wherein the drug nanocrystals are embedded in the matrix of the excipient [29,36]. We hypothesized that the NCSD would require minimal processing for granulation and generation of OSD. In this paper, we aimed to compare downstream processing of NS and NCSD to develop OSD of DCF.

During the generation of NCSD, the selection of a spray drying solvent system was challenging as DCF precipitated upon addition of water into the organic phase. The solid content and drug loading were optimized to 0.5% *w*/*w* and 40% *w*/*v* to obtain a stable feed solution. MAN was selected as a crystallization-inducing matrix excipient due to higher % Yield, low moisture content and good flow properties. In NCSD1, both MAN and DCF were obtained in crystalline Form as confirmed from DSC and characteristics peaks observed in PXRD. Other excipients in the category of sugars, sugar alcohols and amino acids were also explored to generate NCSD. However, the % yield, % moisture content and flow properties of these prototypes hampered their suitability for further development. NCSD1 was blended with extra granular excipients and filled into size 0 capsules.

For comparison, NS of DCF was prepared by wet media milling in SLS- and HPC-containing media. In 6 h, DCF nanocrystal size in NS was obtained similar to that in NCSD1. NS1 required two steps to formulate OSD: (i) drying of nanosuspension and (ii) physical mixing of granules with excipients. An additional unit process for drying was performed using top-spray FBP. Wet granulation of Pearlitol SD 200 and Celphere 203 was performed using 5% *w*/*v* drug loading of NS1. The minimum batch size produced was 250 g to maintain proper fluidization; hence, a large amount of NS was required to produce sufficient quantity of granules. The role of process parameters in FBP was also studied on granulation. Increment in spray rate and inlet temperature resulted in aggregated particles (Figure 4) and reduction in % Yield, respectively, in both G1 and G2 granules. Celphere^®^ 203 showed over granulation with a slight increase in granulation time (Figure 5).

The impact of downstream processing on dissolution was investigated using a discriminatory dissolution medium. A lower % of DCF dissolved from G1 and G2 capsules (52.68% and 48.37%, respectively) as compared to NCSD1 capsules (65.13%). This signaled towards an increase in DCF nanocrystal size upon downstream processing in the case of G1 and G2 capsules (Figure 8). Several literature reports suggest that there are chances of crystal growth during drying of nanosuspension which eventually decrease the drug dissolution rate [60]. Additionally, the nature of granulating substrate had an impact on the dissolution profile, as dissolution rate of G1 capsules was higher as compared to G2 capsules. This was mainly due to the water insoluble nature of MCC, which prevents drug release from granules unlike Pearlitol^®^ that is water soluble [63,64].

For commercial success of a drug product, a cost effective downstream process is required [65]. The cost analysis showed that NanoCrySP technology required INR 183,683 for downstream processing which was 80% lower than the cost for conversion of NS into OSDs (Table 4; Table 5). The extra unit process necessary for drying of nanosuspension was a major contributor to substantial increase in cost of downstream processing (phase 2). The evaluation of process efficiency parameters such as number of steps to convert nanocrystals to OSDs, solid state of final product, dissolution and cost for downstream processing (phase 2 cost) were compared for both the processes.

A comparison of ratings and scores was performed using an innovation radar tool wherein the radar showcased different quantifiable parameters of both the technologies all-together. The process with radar closer to the periphery of the circle is considered more efficient. The innovation radar tool showed that the area covered by NanoCrySP technology was larger as compared to the conventional approach (Figure 9).

It is necessary to develop a commercially profitable downstream process for nanocrystals along with maintaining dissolution benefits obtained from particle size reduction. NanoCrySP technology displayed promising outcomes—one-step process, generate nanocrystals in powder form, physical mixing with excipients sufficient for capsule filling, rapid dissolution and minimal risk of crystal growth. Therefore, NanoCrySP technology can be employed for the development of nanocrystal-based products in a rapid and cost-efficient manner.

## 5. Conclusions

The comparison of bottom-up spray drying-based NanoCrySP technology and the top-down wet media milling method followed by granulation using FBP was executed to identify an efficient process for the generation of nanocrystal-based OSDs. NanoCrySP technology is a one-step process that produces nanocrystals directly in powder form and consequently, physical mixing with excipients was sufficient to formulate OSDs. On the other hand, nanosuspension generated using media milling and an additional unit process such as wet granulation or drying was essential for the development of the final product. Different process parameters, ingredient attributes and dosage form specifications were critical factors that had a significant impact on the process, and thus, these divergent aspects should be assessed diligently. Furthermore, discriminatory dissolution studies confirmed that there was an increase in DCF nanocrystal size during downstream processing of nanosuspension, unlike NCSDs. Moreover, NanoCrySP technology is cost efficient as phase 2 cost was 80% lower than the conventional process. Innovation radar analysis assisted further to conclude that NanoCrySP technology was a more efficient downstream process as compared to the conventional process considering different formulation, process and costing parameters. Thus, one-step NanoCrySP technology with industrial scalability can be applied to formulate drug nanocrystal-based OSDs.

## Figures and Tables

**Table 1:** Manufacturing formula of Nanocrystalline Solid Dispersion (NCSD1 based capsules of DCF.

Ingredients	Role	Quantity (%)	Quantity per Capsule (mg)
NCSD1 ^†^	API	25.14	91.75
Pearlitol^®^ SD 200/Celphere^®^ 203 *	Diluent	72.31	263.94
Aerosil^®^ 200	Glidant	0.30	1.08
croscarmellose sodium (CCS)	Superdisintegrant	1.76	6.42
sodium stearyl fumarate (SSF)	Lubricant	0.49	1.80
Total fill weight		100	365

^†^ NCSD of DCF: MAN: SLS with 0.5% solid content and 40% drug loading; ^†,^* actual quantity of NCSD1 to be added = 35 × (100/%Assay), as dose of DCF used was 35 mg; ^†^ quantity of NCSD1 to be taken equivalent to 36.702 mg DCF = (36.702 × 500)/200. * Added to nullify the impact of diluent on dissolution of DCF nanocrystals

**Table 2:** Manufacturing formula of G1 and G2 capsules.

Ingredients	Role	Quantity (%)	Quantity per Capsule for G1 Granules (mg)	Quantity per Capsule for G2 Granules (mg)
G1 granules ^†^	API	97.15	355.70	-
G2 granules ^#^		97.15	-	355.70
Aerosil^®^ 200	Glidant	0.30	1.08	1.08
croscarmellose sodium	Superdisintegrant	1.76	6.42	6.42
sodium stearyl fumarate	Lubricant	0.49	1.8	1.80
Total fill weight		100	365	365

^†^ Granules of Pearlitol^®^ SD 200 prepared using 400 mL of DCF nanosuspension (NS1); ^#^ granules of Celphere^®^ 203 prepared using 400 mL of NS1; actual quantity of granules to be added, a = 35 × (100/%Assay), for 35 mg dose of DCF; quantity of granules to be taken = a × 10, as drug loading of granules was 10% *w*/*w*.

**Table 3:** Size of DCF nanocrystals obtained on wet media milling.

Time (Hours)	D90 (nm)	PDI
2	810.07 ± 88.11	0.79 ± 0.09
4	596.29 ± 151.08	0.76 ± 0.07
6	361.61 ± 11.78	0.52 ± 0.14
8	351.32 ± 7.53	0.53 ± 0.16

**Table 4:** Costing parameters of NanoCrySP technology (NCSD1) with 0.5% *w*/*v* solid content.

Ingredients	Quantity	Rate/kg or Liter (INR)	Cost (INR)
**Phase 1**			
DCF	7.2 kg	1100	7920
MAN	10.35 kg	473	4896
SLS USPNF	0.45 kg	950	428
Purified Water	800 L	10	8000
Isopropyl alcohol	2800 L	275	792,000
Total ingredient			813,244
Spray dryer			600,000
Manpower	INR 320 × 60 days × 2 persons		38,400
Electricity	INR 300 × 60 days		18,000
Phase 1 NCSD1			1,469,644
**Phase 2**			
Capsule filling machine			80,000
100,000 capsules		0.18/capsule	18,000
Aerosil^®^ 200	0.11 kg	625	69
Croscarmellose sodium	0.642 kg	1450	931
Sodium stearyl fumarate	0.18 kg	13,750	2475
Inert excipient (Pearlitol^®^ SD 200)	26.39 kg	1150	30,348
or Celphere^®^ 203	26.39 kg	3023	79,780
Manpower	INR 320 ×3 days × 1 person		960
Electricity	INR 300 × 3 days		900
Analytical			50,000
Phase 2 NCSD1 (Celphere^®^ 203)			233,115
Phase 2 NCSD1 (Pearlitol^®^ SD 200)			183,683

**Table 5:** Different costing attributes for wet media milling approach.

Ingredients	Quantity	Rate/kg or Liter (INR)	Cost (INR)
	Phase 1		
DCF	4.4 kg	1100	4840
HPC EP	0.88 kg	3300	2904
SLS USPNF	0.088 kg	950	84
Purified Water	88 L	10	880
Total ingredient			8708
Wet media mill (dyno mill)			500,000
Manpower	INR 320 × 2 days × 2 persons		1280
Electricity	INR 300 × 2 days		600
Phase 1 NS1			510,588
	Phase 2		
**Wet granulation using NS**			
FBP			750,000
Inert excipient Pearlitol^®^ SD 200 or	37.7 kg	1150	43,355
Celphere^®^ 203	37.7 kg	3023	113,967
Manpower	INR 320 × 8 days × 2 persons		5120
Electricity	INR 300 × 8 days		2400
**Capsule manufacturing**			
Capsule filling machine			80,000
Aerosil 200	0.11 kg	625	68
Croscarmellose sodium	0.642 kg	1450	931
Sodium stearyl fumarate	0.18 kg	13,750	2475
100,000 capsules		0.18/capsule	18,000
Manpower	INR 320 × 3 days × 1 person		960
Electricity	INR 300 × 3 days		900
Analytical			50,000
Phase 2 NS1 (Celphere^®^ 203)			1,024,821
Phase 2 NS1 (Pearlitol^®^ SD 200)			954,209

**Table 6:** Scoring of different parameters of NanoCrySP technology and conventional approach.

Parameters	(a) Weight	(b) NanoCrySP Rating	(c) Conventional Rating	(a × b) Score NanoCrySP	(a × c) Score Conventional
No. of steps	5	5	4	25	20
Intermediate product stability	4	5	1	20	4
DCF nanocrystal size of intermediate product	3	4	4	12	12
Solid state of final product	5	5	5	25	25
Yield (%)	4	3	5	12	20
Time (days) for phase 2	4	5	2	20	8
Powder flow properties	3	5	5	15	15
Particles interaction	3	5	3	15	9
Input materials	3	1	1	3	3
No. of optimization parameters in phase 2	3	5	3	15	9
Dissolution	5	4	2	20	10
Reproducibility	4	4	1	16	4
Phase 2 cost	5	4	1	20	5

## Supplementary Materials

The following are available online at https://www.mdpi.com/1999-4923/12/11/1015/s1, Table S1: Spray drying solvent system was screened with solid content and drug loading in the range of 0.5–2% *w*/*w* and 20–40% *w*/*v*, respectively, for generation of ternary NCSDs; Section 1: The mechanism of generation of DCF nanocrystals in presence of MAN was investigated using calculation of miscibility parameters and mDSC study; Table S2: Solubility parameters were calculated using Hildebrand, Hoy and Hoftyzer, and Van Krevelen methods to understand miscibility of DCF and MAN; Figure S1: DSC heating curves showed decrease in ΔCp value of DCF with gradual increase in MAN concentration in the physical mixture of DCF and MAN. Table S3: Trend in ΔCp values of DCF with increase in concentration of mannitol in PM depicted that MAN provided heterogeneous nucleation sites for generation of DCF nanocrystals; Table S4: This table mentions the crystallization-inducing excipients scrutinized for generation of ternary NCSDs, % yield, flow properties of various prototype NCSDs were also compiled, Table S5: Stabilizers used for generation of ternary NCSDs were assessed using dispersibility studies wherein the wetting time of each stabilizer system has been mentioned as mean ± SD; Table S6: Size of DCF nanocrystals in various prototype NCSDs was calculated using Zetasizer^®^. D90 and PDI of each NCSD prototype are mentioned as mean ± SD; Table S7: % Moisture content of different prototype NCSDs, as measured using Karl Fischer method, was reported as mean ± SD; Table S8: Bulk density, tapped density, Hausner ratio, Carr’s index and angle of repose of NCSD1 capsule blend, G1 capsule blend and G2 capsule blend are compiled; Section 2: Solubility of DCF in different buffer systems ranging from pH 1.2 to 6.8 were compiled which helped to develop discriminatory dissolution media; Section 3: Development of size-based discriminatory dissolution method considering various optimization parameters; Table S9: Examination of size-based discriminatory dissolution method by varying dissolution medium, rpm, dissolution media volume and % SLS; Figure S2: Discriminatory dissolution method was compared using three different nanosuspensions with variable DCF D90 values; Dissolution profiles are included, Section 4: The probable reasons for lower % dissolved with time in the case of G2 granules as compared to G1 granules in discriminatory dissolution medium were discussed by supporting literature; Figure S3: Overlay of X-ray diffractograms of 0, 30 and 90 days stability samples of NCSD1 showing characteristic peaks of Form II of DCF; Table S10: Particle size analysis of NCSD1 up to 90 days using Zetasizer^®^ proved no significant increase in size of DCF nanocrystals on storage; Table S11: Particle size distribution of 5, 10 and 20 days stability samples of NS1 at 25 °C were reported (mean ± SD); Figure S4: Dissolution profiles of 0, 15, 30 and 90 days stability samples of NCSD1 and physical mixture were depicted to examine crystal growth on storage; Figure S5: Dissolution profiles of stability samples of NS1 on 0, 5, 10 and 20 days and microsuspension were compared to analyze if dissolution changes over a 20 days time period. Section 5: Effect of solid content of NCSD on cost of NanoCrySP technology was discussed to understand whether the change in solid content of NCSD had any significant impact on Phase 1 cost; Table S12: Cost (INR) for NanoCrySP technology with 2% and 5% *w*/*v* solid content was calculated. The increment in cost with increase in solid content was discussed; Table S13: The effect of inert excipient used during Phase 2 was analyzed by calculating cost for Pearlitol^®^ SD 200 and Celphere^®^ 203; Table S14: Different characteristics of NanoCrySP technology and conventional approach were discussed; Table S15: Rating and weightage scale used to quantify parameters was discussed; Table S16: Grading of score decision for parameters was mentioned, from 0 to 25; Table S17: Classification of rating for variable outputs for different parameters was performed which helped to designate rating for a particular attribute of NanoCrySP technology and conventional approach.

## References

[1] Gao L., Liu G., Ma J., Wang X., Zhou L., Li X., Wang F. (2013). Application of drug nanocrystal technologies on oral drug delivery of poorly soluble drugs. Pharm. Res..

[2] Möschwitzer J.P. (2013). Drug nanocrystals in the commercial pharmaceutical development process. Int. J. Pharm..

[3] Wu W., Nancollas G.H. (1998). A new understanding of the relationship between solubility and particle size. J. Solut. Chem..

[4] Mohana Raghava Srivalli K., Mishra B. (2015). Drug nanocrystals: Four basic prerequisites for formulation development and scale-up. Curr. Drug Targets.

[5] Chen H.K.C., Yang X., Chang X., Gao J. (2011). Nanonization strategies for poorly water-soluble drugs. Drug Discov. Today.

[6] Sinha B., Müller R.H., Möschwitzer J.P. (2013). Bottom-up approaches for preparing drug nanocrystals: Formulations and factors affecting particle size. Int. J. Pharm..

[7] Gao L., Zhang D., Chen M. (2008). Drug nanocrystals for the formulation of poorly soluble drugs and its application as a potential drug delivery system. J. Nanoparticle Res..

[8] Shegokar R., Müller R.H. (2010). Nanocrystals: Industrially feasible multifunctional formulation technology for poorly soluble actives. Int. J. Pharm..

[9] Verma S., Gokhale R., Burgess D.J. (2009). A comparative study of top-down and bottom-up approaches for the preparation of micro/nanosuspensions. Int. J. Pharm..

[10] De Waard H., Hinrichs W., Frijlink H. (2008). A novel bottom–up process to produce drug nanocrystals: Controlled crystallization during freeze-drying. J. Control. Release.

[11] Chan H.-K., Kwok P.C.L. (2011). Production methods for nanodrug particles using the bottom-up approach. Adv. Drug Deliv. Rev..

[12] Müller R.H., Jacobs C., Kayser O. (2001). Nanosuspensions as particulate drug formulations in therapy: Rationale for development and what we can expect for the future. Adv. Drug Deliv. Rev..

[13] Shegokar R., Singh K.K., Müller R.H. (2011). Nevirapine nanosuspension: Comparative investigation of production methods. Nanotechnol. Dev..

[14] Chin W.W.L., Parmentier J., Widzinski M., Tan E.H., Gokhale R. (2014). A brief literature and patent review of nanosuspensions to a final drug product. J. Pharm. Sci..

[15] Malamatari M., Somavarapu S., Taylor K.M., Buckton G. (2016). Solidification of nanosuspensions for the production of solid oral dosage forms and inhalable dry powders. Expert Opin. Drug Deliv..

[16] Patel M., Shah A., Patel N., Patel M., Patel K. (2011). Nanosuspension: A novel approach for drug delivery system. J. Pharm. Sci. Bio-Sci. Res..

[17] Goel S., Sachdeva M., Agarwal V. (2019). Nanosuspension technology: Recent patents on drug delivery and their characterizations. Recent Pat Drug Deliv Formul.

[18] Ali H.S., Hanafy A.F., Alqurshi A. (2019). Engineering of solidified glyburide nanocrystals for tablet formulation via loading of carriers: Downstream processing, characterization, and bioavailability. Int. J. Nanomed..

[19] Chaubal M.V., Popescu C. (2008). Conversion of nanosuspensions into dry powders by spray drying: A case study. Pharm. Res..

[20] Salazar J., Ghanem A., Müller R.H., Möschwitzer J.P. (2012). Nanocrystals: Comparison of the size reduction effectiveness of a novel combinative method with conventional top-down approaches. Eur. J. Pharm. Biopharm..

[21] Van Eerdenbrugh B., Froyen L., Van Humbeeck J., Martens J.A., Augustijns P., Van Den Mooter G. (2008). Alternative matrix formers for nanosuspension solidification: Dissolution performance and X-ray microanalysis as an evaluation tool for powder dispersion. Eur. J. Pharm. Sci..

[22] Dolenc A., Kristl J., Baumgartner S., Planinšek O. (2009). Advantages of celecoxib nanosuspension formulation and transformation into tablets. Int. J. Pharm..

[23] Lee J. (2003). Drug nano-and microparticles processed into solid dosage forms: Physical properties. J. Pharm. Sci..

[24] Gulsun T., Gursoy R.N., Oner L. (2011). Design and characterization of nanocrystal formulations containing ezetimibe. Chem. Pharm. Bull..

[25] Van Eerdenbrugh B., Van den Mooter G., Augustijns P. (2008). Top-down production of drug nanocrystals: Nanosuspension stabilization, miniaturization and transformation into solid products. Int. J. Pharm..

[26] van der Aalst W.M., De Medeiros A.A., Weijters A., Dustdar S., Fiadeiro J.L., Sheth A.P. (2006). Process Equivalence: Comparing Two Process Models Based on Observed Behavior BPM Heidelberg, Germany.

[27] Statistical Analysis in Method Comparison Studies—Part One. https://acutecaretesting.org/en/articles/statistical-analysis-in-method-comparison-studies-part-one.

[28] Nekkanti V., Pillai R., Venkateshwarlu V., Harisudhan T. (2009). Development and characterization of solid oral dosage form incorporating candesartan nanoparticles. Pharm. Dev. Technol..

[29] Shete G., Bansal A.K. (2016). NanoCrySP technology for generation of drug nanocrystals: Translational aspects and business potential. Drug Deliv. Transl. Res..

[30] Bansal A.K., Dantuluri A.K., Shete G., Pawar Y. (2018). Nanocrystalline Solid Dispersion Compositions. 2018. Patent.

[31] Conaghan P.G. (2012). A turbulent decade for NSAIDs: Update on current concepts of classification, epidemiology, comparative efficacy, and toxicity. Rheumatol. Int..

[32] Bhala N., Emberson J., Merhi A., Abramson S., Arber N., Baron J., Bombardier C., Cannon C., Farkouh M., FitzGerald G. (2013). Vascular and upper gastrointestinal effects of non-steroidal anti-inflammatory drugs: Meta-analyses of individual participant data from randomised trials. Lancet.

[33] Schneider V., Lévesque L.E., Zhang B., Hutchinson T., Brophy J.M. (2006). Association of selective and conventional nonsteroidal antiinflammatory drugs with acute renal failure: A population-based, nested case-control analysis. Am. J. Epidemiol.

[34] Desjardins P.J., Olugemo K., Solorio D., Young C.L. (2015). Pharmacokinetic properties and tolerability of low-dose SoluMatrix diclofenac. Clin. Ther..

[35] Llinas A., Burley J.C., Box K.J., Glen R.C., Goodman J.M. (2007). Diclofenac solubility: Independent determination of the intrinsic solubility of three crystal forms. J. Med. Chem..

[36] Bhatt V., Shete G., Bansal A.K. (2015). Mechanism of generation of drug nanocrystals in celecoxib: Mannitol nanocrystalline solid dispersion. Int. J. Pharm..

[37] Lai F., Sinico C., Ennas G., Marongiu F., Marongiu G., Fadda A.M. (2009). Diclofenac nanosuspensions: Influence of preparation procedure and crystal form on drug dissolution behaviour. Int. J. Pharm..

[38] Pireddu R., Sinico C., Ennas G., Marongiu F., Muzzalupo R., Lai F., Fadda A.M. (2015). Novel nanosized formulations of two diclofenac acid polymorphs to improve topical bioavailability. Eur. J. Pharm. Sci..

[39] Food Drug Administration (2017). Q3C-Tables and List Guidance for Industry.

[40] Kaur A., Parmar P.K., Bansal A.K. (2019). Evaluation of different techniques for size determination of drug nanocrystals: A case study of celecoxib nanocrystalline solid dispersion. Pharmaceutics.

[41] Carr R.L. (1965). Evaluating flow properties of solids. Chem. Eng..

[42] Loehnert S. (2010). About statistical analysis of qualitative survey data. Qual. Reliab. Eng. Int..

[43] Sandelowski M., Voils C.I., Knafl G. (2009). On quantitizing. J. Mix. Methods Res..

[44] Srnka K.J., Koeszegi S.T. (2007). From words to numbers: How to transform qualitative data into meaningful quantitative results. Schmalenbach Bus Rev..

[45] Morris L. (2008). Innovation metrics: The innovation process and how to measure it. An InnovationLabs White Paper.

[46] Becher R.-D., Schlünder E.-U. (1998). Fluidized bed granulation—the importance of a drying zone for the particle growth mechanism. Chem. Eng. Process..

[47] Gao J.Z., Jain A., Motheram R., Gray D., Hussain M. (2002). Fluid bed granulation of a poorly water soluble, low density, micronized drug: Comparison with high shear granulation. Int. J. Pharm..

[48] Kleinebudde P. (1997). The crystallite-gel-model for microcrystalline cellulose in wet-granulation, extrusion, and spheronization. Pharm. Res..

[49] Murdande S.B., Shah D.A., Dave R.H. (2015). Impact of nanosizing on solubility and dissolution rate of poorly soluble pharmaceuticals. J. Pharm. Sci..

[50] Braig V., Konnerth C., Peukert W., Lee G. (2019). Enhanced dissolution of naproxen from pure-drug, crystalline nanoparticles: A case study formulated into spray-dried granules and compressed tablets. Int. J. Pharm..

[51] Sun D.D., Wen H., Taylor L.S. (2016). Non-sink dissolution conditions for predicting product quality and in vivo performance of supersaturating drug delivery systems. J. Pharm. Sci..

[52] (1997). Guidance for Industry: Dissolution Testing of Immediate Release Solid Oral Dosage Forms.

[53] Shah V.P., Tsong Y., Sathe P., Liu J.-P. (1998). In vitro dissolution profile comparison—Statistics and analysis of the similarity factor, f2. Pharm. Res..

[54] Singh S.K., Srinivasan K., Gowthamarajan K., Narayan G. (2014). Development and validation of discriminatory dissolution procedure for poorly soluble glyburide. Asian J. Pharm..

[55] Ashokraj Y., Daroi A., Gupta R., Khanolkar A., Kulkarni A., Laud S., Pokale M., Shedge S., Date P. (2016). Discriminatory dissolution method development and validation of etoricoxib tablets. Dissolut. Technol..

[56] Pawar H.A., Joshi P.R. (2014). Development and validation of a discriminating in vitro dissolution method for oral formulations containing satranidazole. Int. J. Spectrosc..

[57] Bhakay A., Davé R., Bilgili E. (2013). Recovery of BCS Class II drugs during aqueous redispersion of core–shell type nanocomposite particles produced via fluidized bed coating. Powder Technol..

[58] Kasi V., Tang X. (2005). Design attributes and performance outcomes: A framework for comparing business processes. SAIS 2005 Proceedings.

[59] Arroniz I., Wolcott R.C., Sawhney M. (2006). The 12 different ways for companies to innovate. Engineering Management Review, IEEE.

[60] Bose S., Schenck D., Ghosh I., Hollywood A., Maulit E., Ruegger C. (2012). Application of spray granulation for conversion of a nanosuspension into a dry powder form. Eur. J. Pharm. Sci..

[61] Beirowski J., Inghelbrecht S., Arien A., Gieseler H. (2011). Freeze-drying of nanosuspensions, 1: Freezing rate versus formulation design as critical factors to preserve the original particle size distribution. J. Pharm. Sci..

[62] Figueroa C.E., Bose S. (2013). Spray granulation: Importance of process parameters on in vitro and in vivo behavior of dried nanosuspensions. Eur. J. Pharm. Biopharm..

[63] Berggren J., Alderborn G. (2001). Drying behaviour of two sets of microcrystalline cellulose pellets. Int. J. Pharm..

[64] Kállai N., Luhn O., Dredán J., Kovács K., Lengyel M., Antal I. (2010). Evaluation of drug release from coated pellets based on isomalt, sugar, and microcrystalline cellulose inert cores. AAPS PharmSciTech.

[65] Johannesson M., O’Brien B.J. (1998). Economics, pharmaceuticals, and pharmacoeconomics. Med. Decis. Mak..

