# Supplementary material for: Comparison of Downstream Processing of Nanocrystalline Solid Dispersion and Nanosuspension of Diclofenac Acid to Develop Solid Oral Dosage Form
Source: Pharmaceutics. 2020 Oct 23;12(11):1015. doi: 10.3390/pharmaceutics12111015 (PMC7690810; doi:10.3390/pharmaceutics12111015)
Supplement: Supplementary file 1 [file pharmaceutics-12-01015-s001.pdf]

pharmaceutics

Supplementary Materials: Comparison of Downstream Processing of Nanocrystalline Solid Dispersion and Nanosuspension of Diclofenac to Develop Solid Oral Dosage Form

Sanika Jadhav, Amanpreet Kaur and Arvind Kumar Bansal

Table S1. Screening of spray drying solvent system for generation of NCSDs.

Solvent system	Ratio	(%) Solid Content	% Drug Loading	Observation
Water	Alone	-	-	2 mg is insoluble in 10 mL
Methanol	Alone	-	-	up to 200 mg is soluble in 7 mL (7 mL methanol: 3 mL water)
Acetone	Alone	-	-	up to 300 mg is soluble in 7 mL (7 mL acetone: 3 mL water)
Ethanol	alone	-	-	up to 100 mg is soluble in 7 mL (7 mL ethanol: 3 mL water)
Methanol : water	7:03	0.5<br>1<br>1.5<br>2	30	Particles started settling down after 5 min
Acetone : water	7:03	0.5<br>1<br>1.5<br>2	30	Particles started settling down after 10 min
Ethanol : water	7:03	0.5<br>1<br>1.5<br>2	30	Particles started settling down after 10 min
Acetonitrile : water	7:03	0.5	30	Particles started settling down after 10 min thus further drug loadings were not studied
THF: water	7:03	0.5<br>1<br>1.5<br>2	30	Solubility is much better compared to all other solvents but started precipitating after 30 min
IPA: THF: water	6:01:03<br>5:02:03<br>4:03:03	0.5	20	Better results compared to 30% D.L. Particles started settling down after 30 min
IPA: Dioxane: Water	5:02:03	0.5<br>1<br>1.5<br>2	30	Better results compared to IPA: THF: Water combination<br>Particles started settling down after 40 min
Methanol: Ethyl acetate: water	6:01:03	0.5	30	Particles started settling down after 10 min thus further drug loadings were not studied

Methanol: Ethyl acetate: water	4:03:03	0.5	30	Particles started settling down after 10 min thus further drug loadings were not studied
IPA: Ethyl acetate: water	4:03:03	0.5	30	Particles started settling down after 10 min<br>Thus further drug loadings were not studied
Ethyl acetate: IPA: water	6:01:03	0.5	30	Immiscible
	5:02:03	0.5	30	
IPA: Water	8:02	0.5	40	Soluble and stable system for 4–5 h
	8:02	1		
	9:01	1		Started precipitating after 2 h
Methanol: Water	8:02	1	40	
	9:01			Started precipitating after 1 h
Acetone: Water	8:02	1	40	Started precipitating after 10 min
	9:01			Started precipitating immediately

1. Mechanistic understanding of crystallization behavior of DCF

1.1. Calculation of Miscibility parameter

Miscibility parameters (δ_t) for DCF and MAN were calculated from their chemical structures using Hildebrand, Hoy and Hoftyzer, and Van Krevelen methods. The δ_t values of DCF and mannitol (MAN) were found to be 24.24 MPa$^{1/2}$ and 38.64 MPa$^{1/2}$ respectively as shown in Table S1. A difference of less than 7 MPa$^{1/2}$ indicates miscibility between APIs and MAN while compounds with δ_t difference of more than 7 MPa$^{1/2}$ are likely to be immiscible [1]. The difference in δ_t values of DCF and MAN was found to be 14.4 MPa$^{1/2}$ which indicated immiscibility between them.

Table S2. Miscibility parameter values for DCF and MAN.

Samples	Hildebrand	Hoftyzer and Van Krevelen	Hoy	Average (δ_t) MPa½
DCF	23.73	23.32	25.66	24.24
MAN	38.17	39.09	38.66	38.64

1.2. Investigation for plasticization or heterogeneous nucleation using mDSC

The mechanism of generation of DCF nanocrystals in the presence of crystallization inducing excipient i.e., plasticization or heterogeneous nucleation or both, was investigated using modulated differential scanning calorimetry (mDSC). mDSC analysis of physical mixtures of DCF and MAN in varying ratios were carried out in duplicates. The plasticization effect can be confirmed if there is a decrease in T_g of DCF with an increasing concentration of MAN while a decrease in the heat capacity (ΔCp) of amorphous form of DCF with an increment of MAN in the physical mixture supports heterogeneous nucleation mechanism [1].

Heat-cool-heat (HCH) protocol was used wherein both DCF and MAN were first melted by heating sample up to 190 °C at a heating rate of 20 °C/min followed by rapid cooling in the second cycle. In the third cycle, ± 0.80 °C modulation amplitude for every 60 s applied and then the sample was heated up to 190 °C at a heating rate of 2 °C/min to observe glass transition temperature (T_g) of generated amorphous form of DCF. Representative heating curves of the third cycle were reported in Figure S1. Table S3 showed that with an increase in the proportion of MAN, ΔCp of amorphous form of DCF has been decreased significantly and change in T_g was subtle as compared to the 100% amorphous DCF. This proved that heterogeneous nucleation was responsible for the generation of

DCF nanocrystals embedded in the ternary NCSDs. Also, the percent amorphous DCF form was decreased as MAN concentration increased.

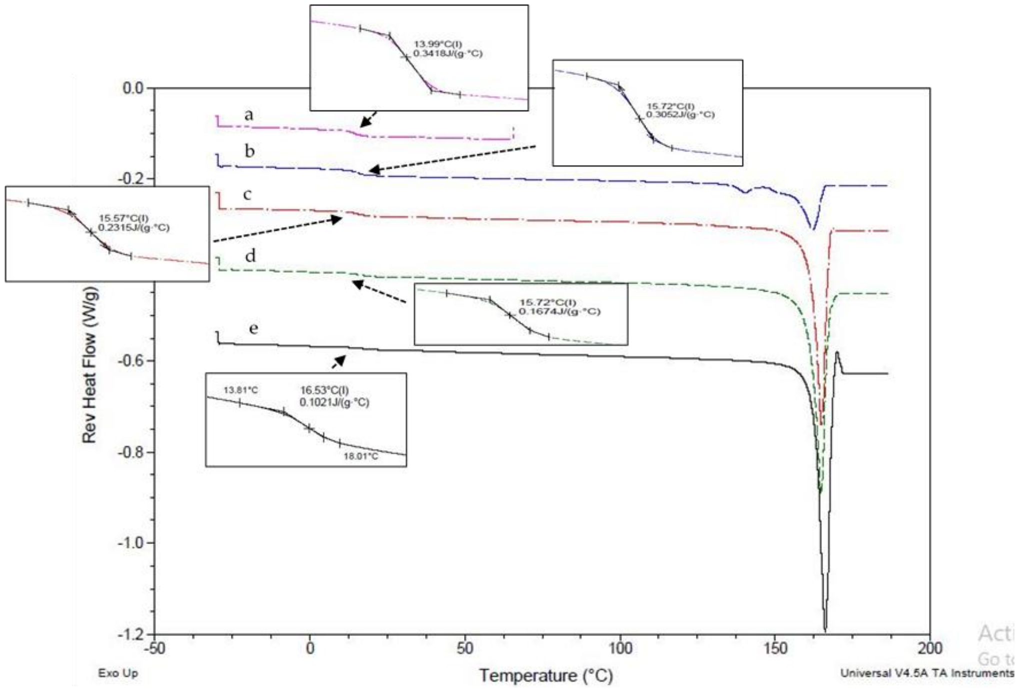

Figure S1. DSC heating curves indicating decrease in ΔCp with increase in MAN concentration. (a)DCF (b) 90% DCF: 10% MAN (c) 70% DCF: 30% MAN (d) 50% DCF: 50% MAN (e) 30% DCF: 70% MAN. Inset shows the zoomed in Tg of DCF in third cycle of mDSC analysis.

Table S3. Trend of ΔCp of DCF with increase in concentration of mannito.

% DCF	% mannitol	T_g (°C)	ΔCp 1	ΔCp 2	ΔCp average	% amorphous DCF
100	0	13.99	0.34	0.34	0.34	100
90	10	15.72	0.31	0.31	0.31	89.30
70	30	15.57	0.23	0.23	0.23	75.89
50	50	15.72	0.17	0.17	0.17	72.29
30	70	16.53	0.10	0.10	0.10	60.96

Table S4. Screening of crystallization inducing excipients for ternary NCSDs generation with 0.5% *w/v* solid content and 40% *w/w* drug loading with 57.5% *w/w* excipient and 2.5% *w/w* SLS.

Excipient class	Excipient name	Remarks
Sugar alcohols	Mannitol (MAN)	Free flowing
	D-sorbitol	Sticky
Sugars	Fructose	Sticky
	Dextrose (DEX)	Relatively less free flowing
	Sucrose (SUR)	Sticky
	Lactose (LAC)	Sticky
	Trehalose (TRE)	Relatively less free flowing
	Maltose monohydrate (MAL)	Relatively less free flowing
Amino acids	Alanine	Free flowing
	Threonine (THR)	Free flowing
	Glycine	DCF precipitated after addition of excipient
	Valine	
	Leucine	
	Isoleucine	

Table S5. Assessment of dispersibility of stabilizers for generation of ternary NCSDs.

Stabilizer	Concentration (%)	Time (Seconds)
SLS	0.05	5
DOSS	0.05	6
Lecithin	0.05	8
HPMC LV E5	0.1	9
HPC	0.1	5
PVP	0.1	19
Poloxamer 407	0.1	10
DOSS+SLS+HPMC LV E5	0.06 + 0.03 + 0.1	7
DOSS+HPMC LV E5	0.06 + 0.1	6
Poloxamer 407+DOSS	0.1 + 0.06	8

Table S6. Size of DCF nanocrystals in prototype NCSDs.

Prototype	Medium	D90 (nm)	PDI
DCF-MAN-SLS	Plain water	953.60 ± 44.66	0.79 ± 0.11
DCF-MAN-SLS (NCSD1)	D.M.*	363.76 ± 21.87	0.51 ± 0.34
DCF-ALN-SLS	D.M.	453.83 ± 74.63	0.58 ± 0.34
DCF-DEX-SLS	D.M.	583.25 ± 316.78	0.78 ± 0.16
DCF-LAC-SLS	D.M.	470.14 ± 237.89	0.57 ± 0.30
DCF-MAL-SLS	D.M.	763.77 ± 267.98	0.74 ± 0.12
DCF-SUR-SLS	D.M.	681.68 ± 173.88	0.89 ± 0.17
DCF-THR-SLS	D.M.	627.75 ± 207.30	0.86 ± 0.14
DCF-TRH-SLS	D.M.	711.13 ± 174.50	0.71 ± 0.18

*Dispersion medium

Table S7. Moisture content of different prototype NCSDs.

Sample	%moisture content
DCF	1.09
NCSD1	0.27
DCF-ALN-SLS	4.56
DCF-THR-SLS	1.08
DCF-DEX-SLS	3.80
DCF-TRE-SLS	5.36
DCF-MAL-SLS	2.76
DCF-LAC-SLS	2.58

Table S8. Various properties of powder blends.

Parameters	NCSD1	G1	G2
Bulk density (g/mL)	0.56	0.59	0.58
Tapped density (g/mL)	0.63	0.69	0.69
Carr's Index	11.19	13.35	16.40
Hausner ratio	1.13	1.15	1.19
Angle of repose	36.30	35.13	36.72
Disintegration time (min)	2.09	2.27	2.48

2. Solubility study of DCF

Apparent solubility of DCF was determined at different pH conditions-—1.2 (HCl buffer), 4.5 (acetate buffer), 5.5 (citrate buffer), and 6.8 (phosphate buffer). The excess amount of DCF was dispersed into each flask containing 10 mL of media in triplicates. The stirring of dispersion was maintained for 72 h using a mechanical shaking bath at 60 rpm and 37 ± 0.5 °C. One millilitre sample was collected from each flask at specified time intervals of 24, 48, and 72 h and filtered through a nylon membrane syringe filter of pore size 0.1 μm. Using acetonitrile in 1:1 (*v/v*) ratio, 500 μL of the filtrate was diluted immediately and samples were analysed using the developed HPLC method. Solubility studies conferred that citrate buffer (pH: 5.5) with 28 μg/mL solubility of DCF, would provide non-sink conditions for dissolution as compared to phosphate buffer (pH: 6.8) having higher solubility of DCF i.e., 320 μg/mL.

3. Screening of discriminatory dissolution medium

This study aimed to develop a discriminatory dissolution medium that showed differentiable dissolution with variable DCF particle size which was essential to detect any change in DCF nanocrystals size during downstream processing. Nanosuspensions with variable D90 values of DCF nanocrystals i.e., NS2: 234 nm, NS3:750 nm and NS4: 1289 nm were prepared according to the procedure mentioned in the Section 2.2.4. A comparison of dissolution profiles of these three nanosuspensions was helpful to develop better discriminatory medium. Table S6 has listed out various dissolution conditions screened for this purpose.

Also, F2 (similarity) factor values calculated by comparing NS2 and NS3 dissolution profiles in the respective medium were mentioned in Table S9. Comparative dissolution profiles of nanosuspensions in different media are given in Figure S2.

Table S9. Screening of API size based discriminatory dissolution medium.

Sr. No.	Buffer	% SLS	Volume (mL)	rpm	F2	Remarks
1	Water	0.1	900	75	86.50	Similar
2	Phosphate buffer pH 6.8	-	900	75	81.75	Similar
3	Citrate buffer pH 5.5	-	900	75	77.71	Similar
4	Citrate buffer pH 5.5	0.1	900	75	71.62	Similar
5	Citrate buffer pH 5.5	-	1000	50	44.77	Dissimilar

Initially, 900 mL water with 0.1% of SLS was used as the medium along with 75 rpm, wherein dissolution profiles of nanosuspensions were overlapped with each other and no discrimination was observed. Solubility studies showed that DCF was more soluble in citrate buffer (pH: 5.5) and phosphate buffer (pH: 6.8) as compared to other media. Thus, these two media were further evaluated to develop size based discriminatory dissolution medium. First, 900 mL phosphate buffer (pH: 6.8) with 75 rpm was tested which showed higher %DCF dissolved with time but was found to be incapable of discriminating dissolution profiles of nanosuspensions. After this, 900 mL of citrate buffer (pH: 5.5) with 75 rpm was evaluated and it had shown slightly variable dissolution profiles of nanosuspension but these profiles were not size based discriminatory. The addition of 0.1% Sodium Lauryl Sulphate (SLS) in this medium resulted in variable dissolution with time of nanosuspensions but did not produce discriminatory dissolution profiles. Thus, SLS did not help to improve discrimination of dissolution profiles based on DCF particle size. The discriminatory dissolution profiles of nanosuspensions were observed when 1000 mL of citrate buffer (pH: 5.5) with sink index 0.8 and 50 rpm was chosen as the discriminatory dissolution medium. With F2 value of 44.77, this

medium helped to show DCF particle size based discrimination in the above-mentioned nanosuspensions.

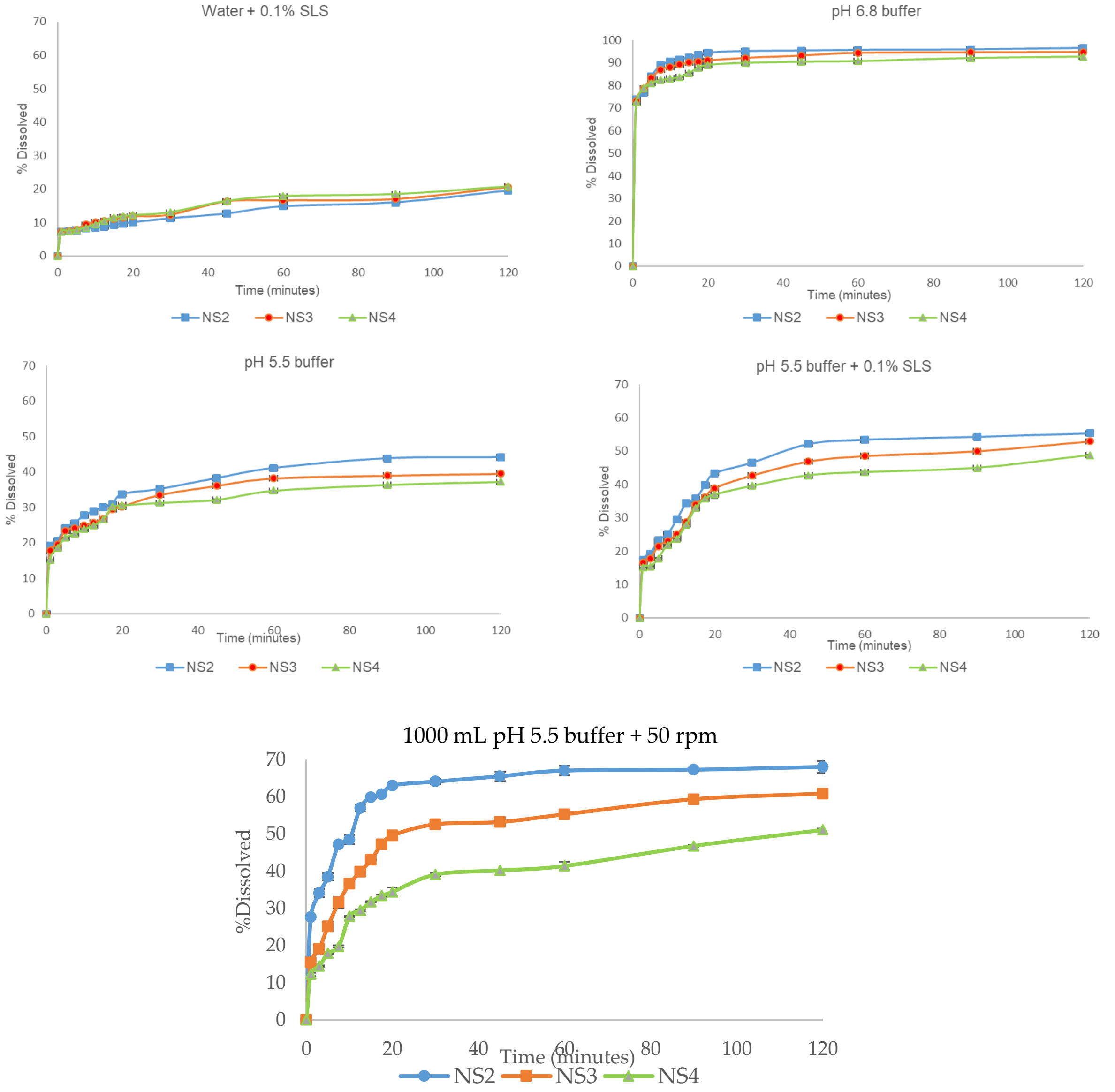

Figure S2. Screening of DCF size based discriminatory dissolution media by comparing three different nanosuspensions having variable DCF D90 values (NS2: 234 nm, NS3:750 nm, and NS4: 1289 nm).

4. Influence of granulating substrate on the dissolution profile

Dissolution with time of G1 capsules (52.68% after 120 min) in discriminatory dissolution medium was higher than that of G2 capsules (48.37% after 120 min). This difference in % DCF dissolved with time signified the impact of excipient properties on drug release. Celphere® 203 which is a grade of MCC doesn't dissolve in water but rather swells to absorb water unlike mannitol based Pearlitol® SD 200 which is water soluble. For drug dissolution, it is necessary to have osmotic pressure difference between the outer and inner part of the granules and this pressure difference may be responsible for the release of the active ingredient which can be explained by capillary pressure theory [2]. In the case of the G1 capsules, this pressure difference was achieved and the active ingredient was released from granules. In contrast, % drug dissolved with time from G2 capsules was slow and less as compared to G1 capsules as the required pressure difference did not build up. As sugar based excipients have higher osmotic activity than MCC, they drove more water into the

system which further increased the dissolution from G1 capsules [3,4]. Another probable reason for lesser % drug dissolution with time of G2 capsules is attributed to crystalline gel model followed by MCC. During wet granulation when MCC comes in contact with water, water destroys crystalline structure of it and MCC converts into a gel. The further drying process results in autohesion to form a stable solid matrix which does not disintegrate easily after coming in contact with water [5].

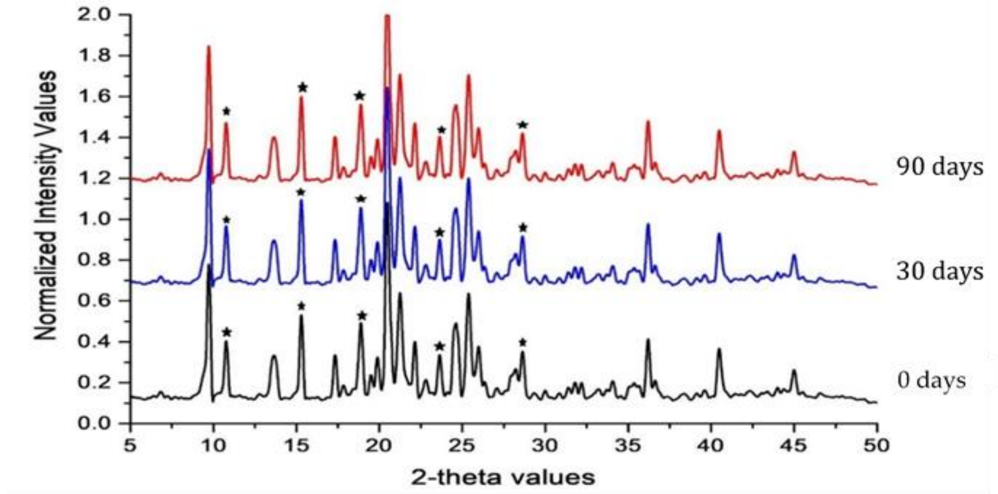

Figure S3. Overlay of X-ray diffractograms of 0, 30 and 90 days stability samples of NCSD1. Characteristic peaks of polymorph II DCF were observed at 2θ values of 10.8°, 15.33°, 18.94°, 24.68 °, and 28.63°. The characteristic peaks of DCF have been marked with symbol *

Table S10. Particle size analysis for stability evaluation of NCSD1 for 90 days.

Days	D90 (nm)	PDI
0	363.76 ± 63.87	0.51 ± 0.10
30	375.41 ± 28.19	0.46 ± 0.26
90	396.32 ± 34.69	0.53 ± 0.16

Table S11. Particle size distribution of 5, 10 and 20 days stability samples of NS1 at 25 °C.

Days	D90 (nm)	PDI
0	369.07 ± 15.47	0.34 ± 0.10
5	380.41 ± 37.84	0.29 ± 0.04
10	384.11 ± 27.44	0.49 ± 0.16
20	403.23 ± 37.01	0.53 ± 0.08

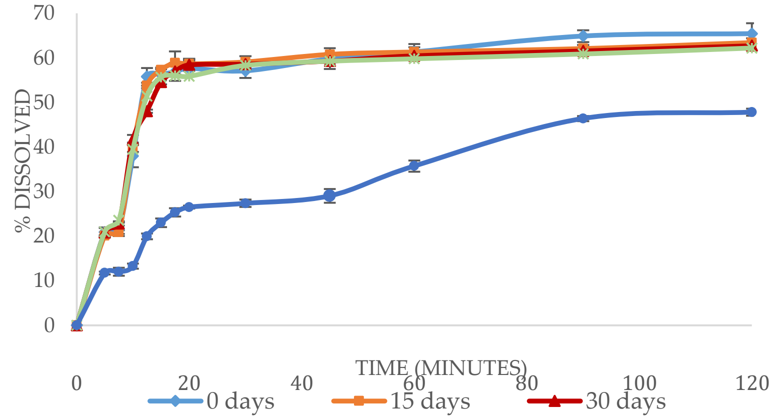

Figure S4. Dissolution profiles of 0, 15, 30 and 90 days stability samples of NCSD1 and physical mixture.

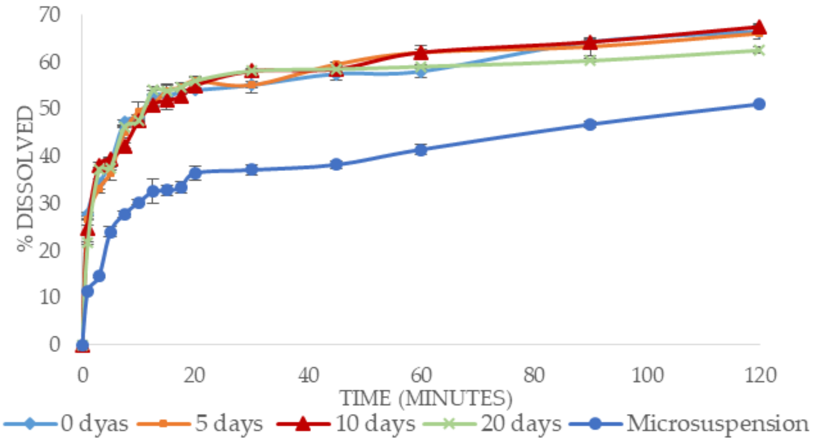

Figure S5. Dissolution profiles of stability samples of NS1 of 0, 5, 10 and 20 days and microsuspension.

5. Effect of solid content of NCSD on cost of NanoCrySP technology

Cost analysis for NCSD with a solid content of 2% *w/v* and 5% *w/v* is mentioned in Table S13 to understand the impact of solid content of NCSD on phase 1 and phase 2 cost. When solid content of NCSD increased from 0.5 to 2 or 5% *w/v* then Phase 1 cost of NanoCrySP technology reduced by 43% or 52%, respectively. With an increment in solid content of NCSD, significant decrease in manpower and electricity cost further reduced the overall phase 1 cost for NanoCrySP technology. However, Phase 2 cost for NCSD with 5% *w/v* solid content did not decrease significantly as compared to 0.5% solid content. This showed that increment in solid content of NCSD did not have a profound impact on downstream processing cost for NanoCrySP technology.

Table S12. Cost (INR) for NanoCrySP technology with 2% *w/v* and 5% *w/v* solid content.

Parameter	For 2% *w/v* solid content	Cost for 2% *w/v* solid content	For 5% *w/v* solid content	Cost for 5% *w/v* solid content
		**Phase 1**		
Total ingredient		2,13,044		93,164
Manpower	INR 320 × 15 days × 2 persons	9600	INR 320 × 6 days × 2 persons	3840
Electricity	INR 300 × 15 days	4500	INR 300 × 6 days	1800
Phase 1 NCSD1		8,27,144		6,98,804
		**Phase 2**		
NCSD1 (Celphere®203)		2,33,115		2,33,115
NCSD 1 (Pearlitol® SD 200)		1,83,683		1,83,683

Table S13. Comparison of costing values (INR) for NanoCrySP technology and conventional approach.

Phases	NS1	NCSD1	Difference (NS1-NCSD1)	% Decrease in cost for NCSD1
		Celphere® 203 as inert excipient		
Phase 1	510,588	1,469,644	−959,056	−187.83
Phase 2	1,024,821	233,115	791,706	77.25
		Pearlitol® SD 200 as inert excipient		
Phase 1	510,588	1,469,644	−959,056	−187.83
Phase 2	954,209	183,683	770,526	80.75

Table S14. Comparative analysis of attributes of NanoCrySP technology and conventional approach.

Attribute	NanoCrySP	Conventional
Approach	Bottom-up	Top-down followed by wet granulation
No. steps required to convert into powder form	One step	Two step
Intermediate product	NCSD	Nanosuspension (NS)
Intermediate product state	Powder	Liquid (aqueous) dispersion
Stability of intermediate product	According to accelerated stability studies, NCSD was stable up to 90 days	NS was stable up to 20 days at 25 °C but relatively less physically stable as compared to NCSD and hence has to be converted into dried form as early as possible
Measurement of DCF nanocrystal size	DCF nanocrystals size embedded in NCSD was directly measured using Zetasizer®	DCF nanocrystals size in granules could not be measured directly and thus had to rely on discriminatory dissolution method to establish relationship w.r.t. nanocrystal size
Yield	Less i.e., around 50–60%	Good, up to 85–95%
Powder characteristics	Free flowing (Carr's Index: 11.19 & angle of repose: 36.29)	Free flowing (Carr's Index: 13.35, 16.40 & angle of repose: 35.13. 36.72)
Particles interaction	No agglomerates were formed	Aggregates of granulated nanosuspension were observed in microscopic images
Time to convert nanocrystals to 100,000 capsules	3 days	11 days
Reproducibility w.r.t % drug release	More reproducible (S.D.: 0.82)	S.D.: 2.88. Thus, less reproducible process
Input materials	Relatively less excipients and stabilizers required	More as excipients and stabilizers were included in both steps
No. of optimization parameters in phase 2	Just physical mixing with excipients was required, not more than 3 parameters to be optimized	As two-step process, wet granulation process parameters along with drug loading of NS, type of dosage form, size of dosage form to be optimized
Dissolution in discriminatory medium	High (65.13%)	Less (48.37%/52.63%)
Environmental concern	Yes due to organic solvent usage	Less as no involvement of organic solvents

Table S15. Rating and weightage scale used to quantify parameters.

Rating/Weightage	Rating Classification	Weightage Categories
1	Not desirable	Not at all important
2	Poor	Slightly important
3	Moderate	Important
4	Good	Fairly important
5	Very good	Most important

Table S16. Score decision for parameters.

Score	Attribute
1 to 5	Very Poor
6 to 10	Poor
11 to 15	Average
16 to 20	Good
21 to 25	Excellent

Table S17. Classification of rating for variable outputs for different parameters.

Parameter/Rating	1	2	3	4	5
No. of steps	More than four	Four	Three	Two	One
Intermediate product	Suspension	Hygroscopic powder	Sticky powder	Poor flowability powder	Free flowing powder
DCF nanocrystal size (nm) of intermediate product	More than 1000 nm	1000–700	500–700	300–500	100–300
Solid state of final product	Amorphous	Crystalline with more than 20% amorphous	Crystalline with 5–20% amorphous	Crystalline with 1–5% amorphous	Crystalline
Yield (%)	0 to 20	20–40	40–60	60–80	80–100
Time (days) for phase 2	More than 12	9 to 12	6 to 9	3 to 6	1 to 3
Powder flow properties	Very poor (sticky)	Poor (Lumps-Hygroscopic)	Fair-passable (Lumps)	Good (Moderately free flowing)	Excellent (Free flowing)
Particle interaction	Significant more than 40% agglomerates and more than 20% aggregates	Moderately 20–40 % agglomerates and 10–20% aggregates	Less amount 10–20% of agglomerates and very less 5–10 % aggregates	Less amount; 5–10% of agglomerates and no aggregates	No aggregates and agglomerates
Input materials	API+ excipients/ stabilizers (more than 50% of total)	API+ excipients/ stabilizers (30–50%)	API+more than 1 or 2 excipients/ stabilizers (10–30%)	API+ stabilizer or API+ excipient (up to 10%)	Only API
No. of optimization parameters in phase 2	More than 10	7 to 9	5 to 7	3 to 5	1 to 3
% drug dissolved in discriminatory medium	Less than 50%	50–55%	55–60%	60–65%	65–75%
Reproducibility (S.D. for disso)	More than 2	1.5 to 2	1 to 1.5	0.5 to 1	0 to 0.5
Cost (for phase 2) (INR)	More than 700,000	500,000–700,000	300,000–500,000	100,000–300,000	Less than 100,000

References

1. Bhatt, V.; Shete, G.; Bansal, A.K. Mechanism of generation of drug nanocrystals in celecoxib: mannitol nanocrystalline solid dispersion. *Int. J. Pharm.* **2015**, 495, 132-9.
2. Berggren, J.; Alderborn, G. Drying behaviour of two sets of microcrystalline cellulose pellets. *Int. J. Pharm.* **2001**, 219, 113-26.
3. Kállai, N.; Luhn, O.; Dredán, J.; Kovács, K.; Lengyel, M.; Antal, I. Evaluation of drug release from coated pellets based on isomalt, sugar, and microcrystalline cellulose inert cores. *AAPS PharmSciTech* **2010**, 11, 383-91.
4. Muschert, S.; Siepmann, F.; Leclercq, B.; Carlin, B.; Siepmann, J. Drug release mechanisms from ethylcellulose: PVA-PEG graft copolymer-coated pellets. *Eur. J. Pharm. Biopharm.* **2009**, 72, 130-7.
5. Kleinebudde, P. The crystallite-gel-model for microcrystalline cellulose in wet-granulation, extrusion, and spheronization. *Pharm. Res.* **1997**, 14, 804-9.
